# Inhibition of p53-Dependent, but Not p53-Independent, Cell Death by U19 Protein from Human Herpesvirus 6B

**DOI:** 10.1371/journal.pone.0059223

**Published:** 2013-03-26

**Authors:** Emil Kofod-Olsen, Janni M. L. Møller, Mariane H. Schleimann, Bettina Bundgaard, Rasmus O. Bak, Bodil Øster, Jacob G. Mikkelsen, Ted Hupp, Per Höllsberg

**Affiliations:** 1 Department of Biomedicine, Aarhus University, Aarhus, Denmark; 2 Institute of Genetics and Molecular Medicine, Cancer Research UK p53 Signal Transduction Laboratories, University of Edinburgh, Edinburgh, Scotland, United Kingdom; Innsbruck Medical University, Austria

## Abstract

Infection with human herpesvirus (HHV)-6B alters cell cycle progression and stabilizes tumor suppressor protein p53. In this study, we have analyzed the activity of p53 after stimulation with p53-dependent and -independent DNA damaging agents during HHV-6B infection. Microarray analysis, Western blotting and confocal microscopy demonstrated that HHV-6B-infected cells were resistant to p53-dependent arrest and cell death after γ irradiation in both permissive and non-permissive cell lines. In contrast, HHV-6B-infected cells died normally through p53-independet DNA damage induced by UV radiation. Moreover, we identified a viral protein involved in inhibition of p53 during HHV-6B-infection. The protein product from the *U19* ORF was able to inhibit p53-dependent signaling following γ irradiation in a manner similar to that observed during infection. Similar to HHV-6B infection, overexpression of U19 failed to rescue the cells from p53-independent death induced by UV radiation. Hence, infection with HHV-6B specifically blocks DNA damage-induced cell death associated with p53 without inhibiting the p53-independent cell death response. This block in p53 function can in part be ascribed to the activities of the viral U19 protein.

## Introduction

Human herpesvirus (HHV)-6B is a ubiquitous herpesvirus in humans with a seroprevalence close to 95% [1], [2]. Infection usually occurs within the first two years of life, after which HHV-6B remains as a lifelong latent infection [3], [4]. Unlike other known herpesviruses, it has been suggested that latency is accompanied by integration of the viral genome into the host cell genome [5]. This has led to establishment of chromosomal integration of HHV-6B into all cells in approximately 1% of individuals [6]. Primary infection is the cause of the common childhood disease exanthem subitum [7] and may give rise to episodes of febrile seizure [8]. The virus reactivates later in life, and might lead to severe and sometimes fatal disease in immune compromised individuals [9]. Moreover, HHV-6B infection has been associated with various diseases, including mesial temporal lope epilepsy [10].

Upon a viral infection, the cell elicits a series of antiviral activities, including activation of the tumor suppressor protein p53. This protein is a key element in controlling the response to different forms of genotoxic stress resulting in the induction of arrest and repair. If the stress persists, this may be followed by programmed cell death through the intrinsic pathway [11]–[15]. The direction of activity of p53 is managed through a wide range of post-translational modifications [16]. During cellular stress such as DNA damage or viral infection, the cell can quickly increase the amount of p53 and try to either repair the damage or induce cell death if the damage is consistent or irreparable. To establish a viral infection, it is therefore of utmost importance for the virus to either prevent the activities of p53 completely or to alter p53 activities to help shape an infection-friendly environment through DNA damage repair mechanisms.

Most herpesviruses have evolved mechanisms to inhibit or alter p53-dependent actions [17]–[20]. One of the most studied systems involves the beta-herpesvirus human cytomegalovirus (HCMV) and its murine counterpart (MCMV). During infection with HCMV, the levels of p53 rise early during infection. This rise in p53 levels is in part due to translocation of the negative inhibitor MDM2 to the cytoplasm where it is degraded [18], [21]. The high level of p53 during early HCMV-infection is transcriptionally active and it is suggested that the virus needs p53 as a transcription factor during the early parts of the infection [22], [23]. Another human beta-herpesvirus that is known to interfere with the p53 network is human herpesvirus (HHV)-6B. Others and we have previously shown that p53 accumulates in the cytoplasm after HHV-6B infection [24]–[26].

Although extensively studied in many other viruses, the regulation and activity of p53 during HHV-6B infection still remain largely unknown. In this report, we show that HHV-6B infection prevents p53-dependent, but not -independent cell death. Moreover, we show that the accumulation of p53 observed during HHV-6B infection can in part be ascribed to the protein product from the *U19* ORF. Expression of this protein inhibited p53 activity and induction of PUMA and apoptosis in a manner similar to that observed during HHV-6B infection.

## Materials and Methods

### Cells and Virus

The human epithelial colon carcinoma cell line HCT116 [27] was a gift from B. Vogelstein and K. W. Kinzler. HCT 116 cells were cultured in McCoy’s 5A medium, the human embryonic kidney cell line 293T (ATCC) was cultured in Dulbecco’s modified Eagle’s medium (DMEM), and the human leukemia T-cell line MOLT3 [28] (a gift from Z. Berneman) was cultured in RPMI medium. All media were supplemented with 10% fetal calf serum, glutamine (0.2 g/L), streptomycin (0.2 g/L), penicillin (0.2 g/L) and HEPES (10 mM). HHV-6B strain PL-1 was propagated in MOLT3 cells, and virus was concentrated by ultracentrifugation, as previously described [29]. The viral titer was determined by TCID_50_ using a thymidine incorporation assay 4 hpi, as previously described [30].

### Induction of Cell Death

Cells exposed to γ radiation were treated with a cesium γ-source for 15 minutes, equivalent to 30 Gy, followed by additional culture for 24 hrs before use. Cells exposed to UV radiation were treated with UV-C light (15 Watt) in minimal amounts of media for 5 sec to 10 min, as indicated, and cultured as indicated. Cells treated with leupeptin (Sigma-Aldrich, Milwaukee, WI), doxorubicin (Sigma Aldrich), or MG132 (Sigma Aldrich) were incubated 24 hrs in 10 µM leupeptin, 0.2 µg/ml doxorubicin, or 10 µM MG132 before use.

### Intracellular ATP Measurements

Cell viability was assessed by measuring intracellular ATP levels using the CellTiter-Glo® Luminescent Cell Viability Assay according to the manufacturers description (Promega). The percentage of dead cells was calculated by the drop in ATP level in treated cells relative to untreated cells. Cells treated with zVAD-fmk (50 µM) (R&D systems, UK) and necrostatin-1 (30 µM) (Sigma Aldrich) were incubated for 1 hr prior to γ or UV irradiation.

### Luciferase Measurements

HCT116 cells wildtype (wt) and U19-S cells were transfected with the WWP-*luciferase* plasmid containing wt WAF1 (p21) promoter with p53 binding sites using the Amaxa transfection system. Luciferase intensity was measured 48 hrs post transfection with the Luciferase 1000 assay system (Promega, Madison, WI, USA) on an Ascent Luminoskan.

### Lentiviral Production

Lentiviral vectors were produced in 293T cells that were seeded in 10-cm dishes (4×10^6^ cells/dish) in DMEM. Twenty-four hrs later, transfections were performed using standard calcium phosphate precipitation of the indicated plasmids into the producer cells (3.75 µg pMD.2G, 3 µg pRSV-Rev, 13 µg pMDGP-Lg/RRE, and 13 µg lentiviral transfer vector). Twenty-four hrs post-transfection the medium was removed and McCoy’s 5A medium was added. Forty-eight hrs post-transfection, the viral supernatant was harvested and filtered through 0.45 µm filters (Sarstedt, Nümbrecht, Germany) and supplemented with 8 µg/ml polybrene (Sigma-Aldrich). In transduction studies, HCT116 cells were seeded in 6-well plates (10^5^ cells/well). Twenty-four hrs later, the cells were transduced with the lentiviral vectors: HCT116 cells were washed with PBS and incubated with crude lentiviral vectors diluted two-fold in McCoy’s 5A medium. The medium was changed 24 hrs post-transduction.

### Microarray Analysis

Affymetrix GeneChip Human Genome U133 plus 2.0 Array was used to examine expression of mRNA from the cells. HCT116 wt cells, HCT116-p53^−/−^ cells, and HHV-6B-infected HCT116 cells (24 hrs) were treated with or without 30 Gy γ radiation and left for additional 4 hrs before total RNA preparation. Total RNA was prepared using RNAzol B (Gibco, Brl, California, USA), as described by the manufacturer. The RNA quality was examined by analysis on a 2100 Bioanalyzer (Agilent) to verify that samples had a 28S/18S ratio higher than 1 and RNA integrity number higher than 7. Total RNA was reverse transcribed and labeled before hybridized to the Human Genome U133 plus 2.0 GeneChip overnight before scanning. For analysis, 128 known p53-response genes and apoptotic marker genes were analyzed using the microarray dataset. The genes lacking significant signals in all cell types/treatment groups were excluded from the analyses. The remaining genes were grouped according to mRNA expression level using hierarchical clustering. Red boxes represent genes with high mRNA expression and blue boxes represent genes with low mRNA expression. The clustering algorithm and heat map was performed using GenePattern software.

### Confocal Microscopy

Cells (10^5^) were transferred to poly-L-lysine coated slides and incubated for 24 hrs followed by fixation in 4% formalin/PBS (pH 7.5). Cells were washed twice in PBS, blocked in 5% BSA/PBS and permeabilized in 0.2% Triton-X 100/PBS. P53 was visualized using a rabbit anti-p53 pAb (FL393) (1∶200) (Santa Cruz Biotechnologies, Santa Crus, CA, USA), mouse anti-p53 mAb (DO-7) (1∶200) (Life Technologies Europe BV) mouse anti-p53-phospho-Ser15 (p-Ser15) mAb (1∶1000) (Cell Signaling Technology, Beverly, MA, USA), p41 was visualized using anti-p41 mAb (1∶400) (Advanced Biotechnologies, Tebu-Bio, Columbia, USA), DR6 was visualized using a rabbit anti-DR6 peptide pAb (1∶200) (GeneScript – custom antibody [25]), active caspase-3 was visualized using a anti-p17-fragment mAb (1∶100) (Abcam, UK). Secondary antibodies were F(ab’)_2_ antibody fragment conjugated with Alexa488 or Alexa546 (1∶400) (Life Technologies). The nucleus was visualized by incubating the cells in DAPI (300 nM)/PBS for 15 min. Imaging was carried out using the 488 nm line of a multi-line argon laser, the 543 nm line of a green helium-neon laser and the 405 nm line of a violet diode laser on a Zeiss LSM710 inverted microscope with a 63x oil-immersion objective.

### Western Blot Analyses

Cells were lysed for 30 min on ice in 1x Lysis Buffer (Cell Signaling) supplemented with 1 mM phenylmethanesulphonylfluoride (PMSF), 5 mM sodium fluoride (NaF) and Complete Mini Protease Inhibitor (Roche Diagnostics Scandinavia AB, Hvidovre, Denmark). Nuclear and cytoplasmic fractions were generated using the Nuclear Extract Kit (Active Motif, Rixensart, Belgium). Proteins were separated on Criterion 12% BIS-TRIS gels (Bio-Rad, Copenhagen, Denmark), blotted to nitrocellulose membranes and visualized with the following antibodies: Rabbit anti-PARP mAb (1∶2000) (Cell Signaling), rabbit anti-GAPDH pAb (1∶1000) (Santa Cruz), mouse anti-RCC1 mAb (1∶1000) (Santa Cruz), mouse anti-p53 mAb (DO-7) (1∶1000) (Life Technologies), rabbit anti-PUMA pAb (1∶1000) (Abcam), mouse anti-p53-phospho-Ser15 (p-Ser15) mAb (1∶1000) (Cell Signaling), mouse anti-p53-phospho-Ser46 (p-Ser46) mAb (1∶1000) (Cell Signaling), mouse anti-HHV-6 mAb (7C7) (1∶1000) (Argene Biosoft, Verniolle, France). Secondary antibodies were horseradish peroxidase-conjugated swine anti-rabbit pAb (P0217) (1∶2000) (DAKO, Glostrup, Denmark), horseradish peroxidase-conjugated rabbit anti-mouse pAb (1∶2000) (P0260, DAKO). Membranes were developed with Chemiluminoscence femto (Pierce, Thermo Scientific, Slangerup, Denmark). All antibodies were used in 5% skimmed milk.

### Cell Cycle Analysis

Cells (10^5^ per sample) were treated with trypsin, washed twice in PBS and resuspended in 200 µl PBS in 96-well microtiter plates. Nuclear Isolation and Stain (NIM-DAPI) (Beckman Coulter Inc., Brea, CA, USA) was added (50 µl) to each sample, and the nuclei were analysed by flow cytometry using the 350 nm ultra-violet laser on a Quanta SC MPL flow cytometer (Beckman Coulter Inc.). The data were analysed using FlowJo software with G2 = 2×G1 constrain and the Dean-Jett-Fox algorithm [31].

### Real-time PCR

RNA was extracted from 10^6^ cells using RNaesy mini kit (Qiagen Nordic, Sollentuna, Sweden). Real time PCR was performed on a Stratagene Mx3005P with the following primers: *TBP*: 5′-GCGGTTTGCTGCGGTAAT-3′ and 5′-GACTGTTCTTCACTCTTGGCT-3; *MDM2*∶5′-ACCACCTCACAGATTCCA-3′ and 5′-CAGGCCAAACAAATCTCC-3′; *PUMA (BBC3)*: 5′-GAGACAAGAGGAGCAGCA-3′ and 5′-ACATGCTGCAGAGAAAGT-3′ (GATCopenhagen, Copenhagen, Denmark). *PUMA* and *MDM2* mRNA levels were determined relative to *TBP* mRNA using the formula: 2^(Ct1/Ct2)^.

## Results

### HHV-6B Inhibits p53-dependent, but not p53-independent, Cell Death

**Figure 1:**
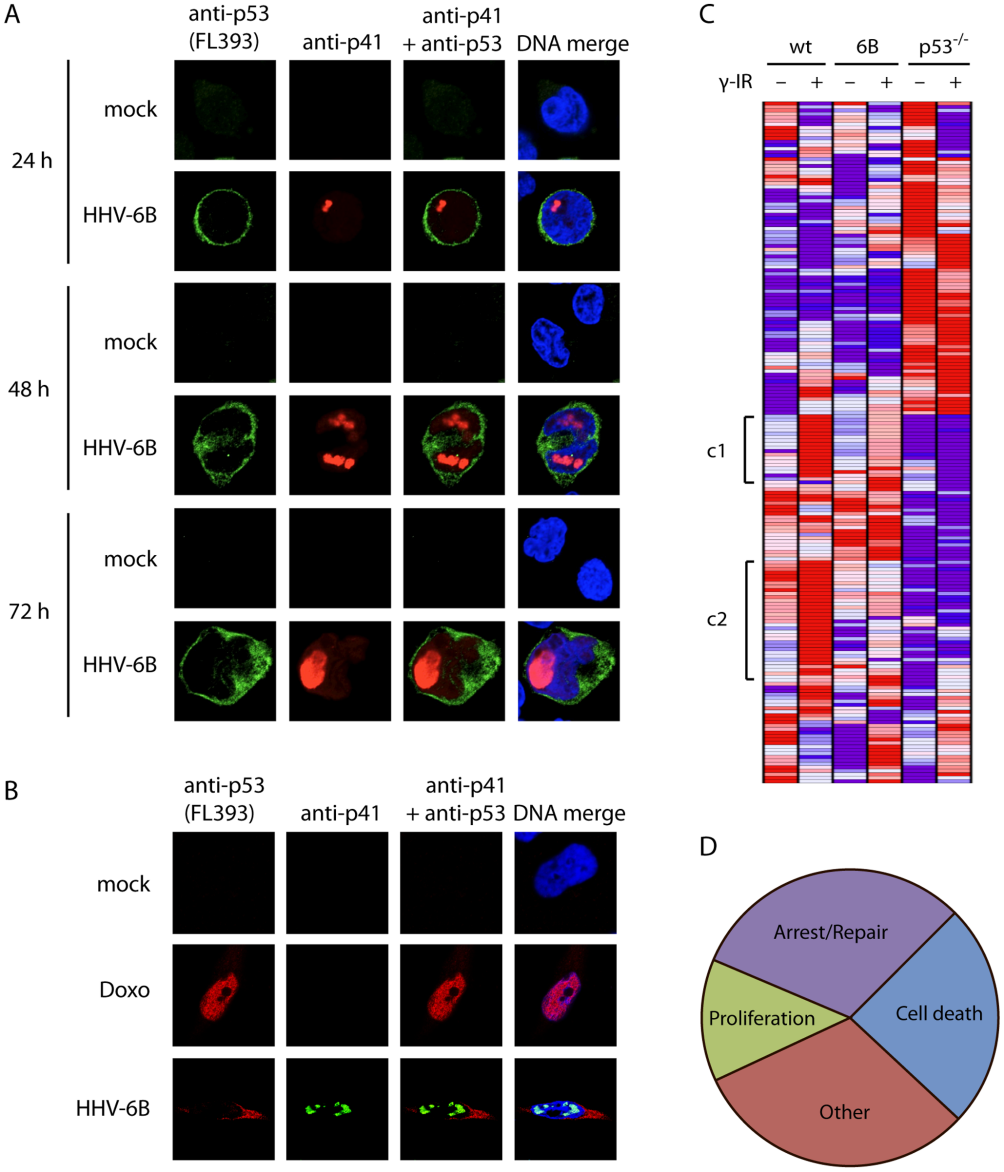
HHV-6B infection leads to cytoplasmic accumulation of p53. (**A**) Confocal microscopy images of MOLT3 cells infected with HHV-6B for 24, 48 or 72 hrs. The cells were analyzed with antibodies against p53 (Alexa488 green), p41 (Alexa546 red) and DAPI (blue). (**B**) Confocal microscopy images of HCT116 cells treated with or without doxorubicine or infected with HHV-6B for 48 hrs, followed by analysis with antibodies against p53 (red), p41 (green) and DAPI (blue). (**C**) Heat-map showing hierarchical cluster analysis on 128 p53-target genes. Red indicates high expression and blue indicates low expression. The symbol [denotes a cluster (c1 and c2) that responds to γ radiation in the wt cells, but with no or impaired response in the HHV-6B-infected cells and p53^−/−^ cells. (**D**) Functional distribution of genes from c1 and c2 in the heat-map analysis in (C). Cell death, 24.1%; Proliferation, 13.1%; Arrest/Repair, 31.1%; Other, 31.1%.

During infection with HHV-6B, p53 is stabilized and accumulates massively in the cytoplasm of infected T cells [24], [26]. It has, however, not been examined if p53 continually stays in the cytoplasm as the infection progresses. We infected MOLT3 cells with HHV-6B (PL-1 strain) for 24, 48, and 72 hrs and analyzed the localization of p53 and the viral DNA-polymerase cofactor protein p41 using confocal microscopy. This analysis showed a cytoplasmic accumulation of p53 during HHV-6B infection at all analyzed time points (Figure 1A). We have previously examined HHV-6B infection of the non-permissive HCT116 cell line [32], [33]. To verify that p53 accumulation was also predominantly cytoplasmic during infection of these cells, we infected them with HHV-6B for 48 hrs and analyzed the localization of p53 with confocal microscopy. This demonstrated that p53 accumulated virtually completely in the cytoplasm, whereas doxorubicin treatment led to nuclear accumulation, as expected (Figure 1B). Thus, in both MOLT3 and HCT116 cells, HHV-6B infection causes p53 stabilization and accumulation. Both of these cell types appear to be infected at a similar rate, but may differ in their ability to shed virus into the supernatant [32], as infection of HCT116 is believed to be non-productive.

Whether or not p53 retains any activity during infection remains an open question. To address this, we performed an analysis on Affymetrix GeneChip Human Genome U133 plus 2.0 Array of expressed genes from HCT116 wt cells and HHV-6B-infected HCT116 cells 4 hrs after treatment with or without 30 Gy γ radiation. In addition, we included HCT116-p53^−/−^ cells to be able to compare the genes affected by HHV-6B with those dependent on p53. From the obtained data set, we selected 128 known p53-response genes (Table S1). Cluster analysis on these genes identified two clusters that were strongly upregulated in the wt cells after γ irradiation, but impaired in their up-regulation to γ radiation in the p53^−/−^ cells and HHV-6B-infected cells (Figure 1C). Half of the genes in these two clusters (55%) are known to be involved in cell death, arrest and/or DNA damage repair (Figure 1D).

**Figure 2:**
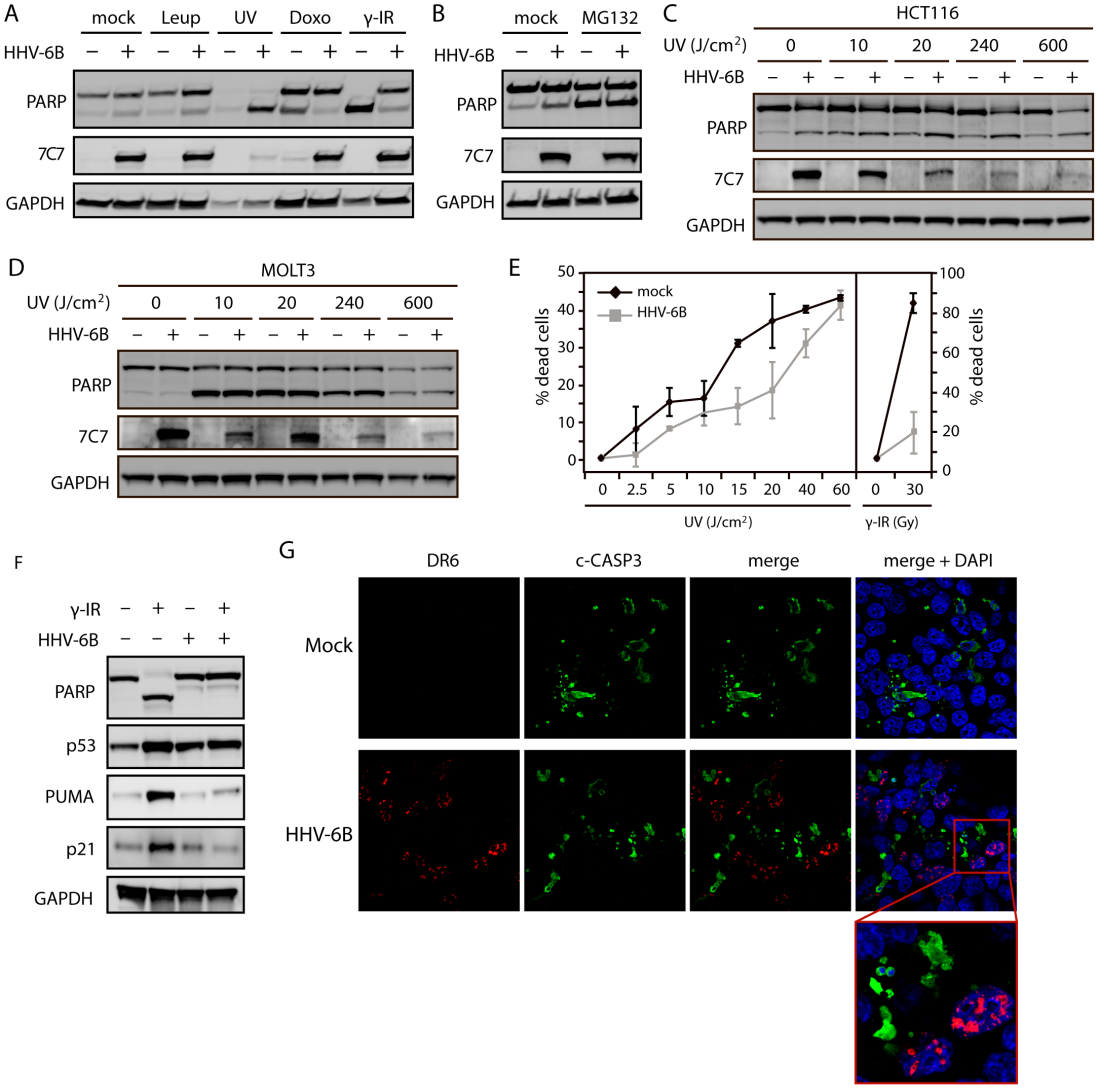
HHV-6B infection rescues cells from p53-dependent apoptosis. (**A & B**) Western blot analysis of PARP cleavage in cells either mock-treated or infected with HHV-6B for 48 hrs. The cells were subsequently treated with mock, leupeptin (10 µM), UV radiation (10 min exposure followed by 4 hrs of incubation), doxorubicin (0.2 µg/ml for 24 hrs), γ radiation (30 Gy followed by 24 hrs of incubation), or MG132 (10 µM). 7C7 was used as infection control and GAPDH was used as loading control. (**C & D**) Western blot analyses with antibodies against PARP, GAPDH, and 7C7 (infection control) on lysates from HCT116 (C) or MOLT3 (D) cells treated with varying doses of UV radiation (10, 20, 240, 600 J/cm^2^ followed by 4 hrs of incubation). (**E**) ATP cell viability assay on MOLT3 cells with or without HHV-6B infection and treated with varying amounts of UV (0, 2.5, 5, 10, 15, 20, 40 and 60 J/cm^2^ followed by 4 hrs of incubation) or γ radiation (0 and 30 Gy followed by 24 hrs of incubation). (**F**) Western blot analyses with antibodies against PARP, p53, PUMA, p21 or GAPDH (loading control) on lysates from HCT116 cells either mock-treated or infected with HHV-6B for 24 hrs followed by γ radiation (30 Gy) and 24 hrs of incubation. (**G**) Confocal microscopy images of HCT116 cells either mock-treated or infected with HHV-6B for 24 hrs, γ-irradiated (30 Gy) followed by 24 hrs of incubation. The cells were analyzed for cleaved caspase-3 (Alexa 488 green), DR6 (Alexa 546 red), and DAPI (blue).

Based on the data obtained from the MicroArray screen, we set out to determine whether the p53-dependent cell death response was also inhibited. We analyzed the ability of HHV-6B to rescue the cells from p53-dependent and –independent cell death induced by different DNA-damaging agents. HCT116 cells were either mock-treated or HHV-6B-infected for 24 hrs followed by treatment with leupeptin (treatment control), ultraviolet light (UV), doxorubicin or γ radiation, and analyzed for poly-ADP-ribose polymerase (PARP) cleavage by Western blotting (Figure 2A). PARP is cleaved into an 89 kDa fragment by effector caspases during apoptosis and is a consistent marker of apoptosis. It is known that γ radiation and doxorubicin treatment lead to a p53-dependent cell death, whereas UV radiation can induce both p53-dependent and –independent cell death [34], [35]. Treatment with UV radiation induced rapid cell death in both infected and uninfected cells as seen by a complete PARP cleavage and by detachment of the cells. In contrast, cells treated with doxorubicin or γ radiation were rescued from cell death if infected by HHV-6B (Figure 2A). To further address whether the infected cells could die from p53-independent death, HCT116 cells were infected with HHV-6B in the presence or absence of the proteasome inhibitor MG132. Both infected and uninfected cells expressed PARP cleavage after MG132 treatment (Figure 2B).

It has previously been reported that HHV-6B infection rescues cells from UV-induced cell death [26]. Since the UV-treatment induced a massive death, as seen by the degraded GAPDH (Figure 2A), we performed UV dose-dependent experiments in both HCT116 (Figure 2C) and MOLT3 cells (Figure 2D). This demonstrated that in neither HCT116 nor MOLT3 cells were HHV-6B infection able to prevent UV-induced cell death. To further address this we analyzed UV-induced cell death quantitatively by measuring intracellular ATP-levels in dose-response measurements using low-dosage UV (Figure 2E). This experiment showed that UV-induced cell death was reduced in HHV-6B-infected cells under treatments below 40 J/cm^2^, but the cells still died in a dose-dependent manner even at very low doses. As expected, the infected cells were rescued from γ radiation-induced death (Figure 2E).

To examine whether or not the infected cells showed any p53 response after stimulation, we treated HCT116 cells with or without HHV-6B for 24 hrs followed by γ radiation (30 Gy) and an additional 24 hrs of incubation. The cells were analyzed by Western blotting with antibodies against PARP and p53 as well as antibodies against the protein product of the p53 response-genes PUMA and p21 (Figure 2F). Both p21 and PUMA protein levels were elevated in the mock-treated cells after γ radiation, but HHV-6B infection of the cells inhibited this upregulation.

To visualize that apoptotic cells were uninfected, HCT116 cells were incubated with or without HHV-6B for 24 hrs, γ-irradiated (30 Gy) followed by incubation for 24 hrs. Confocal microscopy using antibodies against the viral protein DR6 and active caspase-3, and the DNA dye DAPI (Figure 2G) demonstrated that no DR6 positive cells showed apoptotic morphological signs, which was in contrast to the majority of uninfected cells. Cells positive for DR6 were also negative for active caspase-3 (Figure 2G).

### DNA Damage does not Activate p53 in HHV-6B-infected Cells

**Figure 3:**
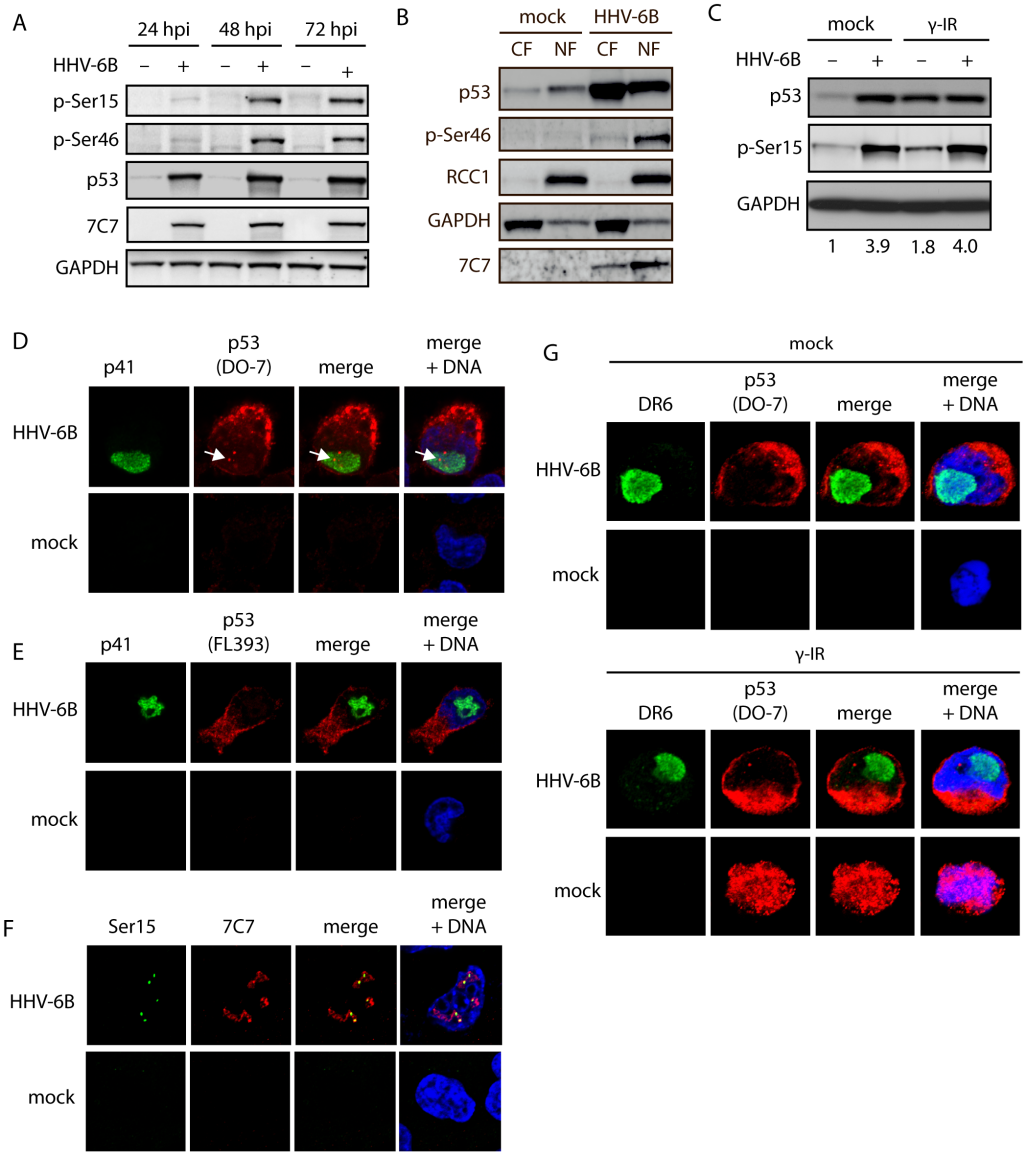
HHV-6B infection leads to nuclear accumulation of phosphorylated p53. (**A**) Western blot analyses of HCT116 cells infected for 24, 48, or 72 hrs and analyzed with antibodies against p53 p-Ser15, p53 p-Ser46, p53, 7C7 (infection control), or GAPDH (loading control). (**B**) Western blot analyses of nuclear (NF) and cytoplasmic (CF) fractions from mock-treated or HHV-6B-infected HCT116 cells (48 hpi). The membranes were stained with antibodies against p53, p53 p-Ser46, 7C7 (infection control), RCC1 (nuclear control), or GAPDH (cytoplasmic control). (**C**) Western blot analyses of HCT116 cells mock-treated or HHV-6B-infected for 24 hrs followed by γ irradiation (30 Gy) and additional incubation for 24 hrs. The membrane was probed with antibodies against p53, p53 p-Ser15, or GAPDH (loading control). Numbers at the bottom of the figure indicate fold induction of Ser15 phosphorylation relative to GAPDH. (**D & E**) Confocal microscopy on MOLT3 cells infected with HHV-6B for 24 hrs followed by staining with antibodies against the viral protein p41 (green), p53 (red) DAPI (blue). The p53 antibodies were either the monoclonal DO-7 (D) or a polyclonal antibody (FL393) (E). Arrows point to nuclear p53 staining. (**F**) Confocal microscopy on MOLT3 cells infected with HHV-6B for 24 hrs followed by staining with antibodies against p53 phospho-Ser20 (green), 7C7 (red) and DAPI (blue). (**G**) Confocal microscopy on MOLT3 cells infected with HHV-6B for 24 hrs, γ-irradiated (30 Gy) for 24 hrs followed by staining with antibodies against the viral protein DR6 (green), p53 (DO-7) (red) DAPI (blue).

We have previously shown that p53 is phosphorylated at Ser15, Ser20, Ser33, and Ser392 during HHV-6B infection [24], [36]. Phosphorylation of Ser46, by HIPK2 and DYRK2 is thought to be essential for p53-induced apoptosis by increasing the binding affinity to promoters of pro-apoptotic genes [37], [38]. To determine if the inability to undergo p53-dependent apoptosis could be due to lack of Ser46 phosphorylation, we infected HCT116 cells for 24, 48, or 72 hrs and analyzed the level of Ser46 phosphorylation. Surprisingly, p53 was excessively phosphorylated at this serine residue during infection (Figure 3A), a phosphorylation that has been suggested to destine the cells for apoptosis [37].

After stress induction, p53 is normally activated and translocated to the nucleus. Thus, HHV-6B-infection may potentially inactivate p53 by sequestering it in the cytoplasm. To determine whether Ser46 phosphorylated p53 was present in the nucleus or the cytoplasm during infection, HCT116 cells were infected and lysed at 48 hpi followed by separation into a cytoplasmic and a nuclear fraction. Importantly, Ser46 phosphorylated p53 was present in the nucleus (Figure 3B), although p53 is predominantly located in the cytoplasm of the infected cells (Figure 1A and 1B). The inability of HHV-6B-infected cells to undergo p53-dependent cell death may therefore not be solely due to p53 sequestration in the cytoplasm.

To address the possibility that p53 could be activated further by DNA damage stimuli during infection, HCT116 cells were either mock-infected or HHV-6B-infected for 24 hrs and γ-irradiated followed by 24 hrs of incubation. Cells were lysed and examined by Western blotting with antibodies against p53 or pSer15 (Figure 3C). The level of Ser15 phosphorylation did not increase further in HHV-6B-infected cells after γ irradiation. This suggests that p53 is not activated further after DNA damage in HHV-6B-infected cells.

To determine if the observed discrepancy between WB fractionations (Figure 2B) and confocal microscopy (Figure 1A and 1B) was due to epitope masking during confocal preparations, MOLT3 cells were either mock-treated or infected with HHV-6B and analyzed by confocal microscopy using FL393 and DO-7, two different anti-p53 antibodies. This analysis showed that FL393 only recognized cytoplasmic p53 during infection, whereas the DO-7 recognized both cytoplasmic and a small fraction of nuclear p53 (Figure 3D and 3E). To address whether this nuclear p53 could represent phosphorylated p53, we also stained mock-treated and HHV-6B-infected cells with anti-phospho-Ser15 antibody. This analysis demonstrated a punctuated, nuclear staining pattern resembling that observed with the DO-7 antibody (Figure 3F).

To rule out that p53 could translocate to the nucleus after DNA-damage stimuli in HHV-6B-infected cells, MOLT3 cells with or without HHV-6B infection were either mock-treated or treated with γ radiation for 24 hrs, followed by confocal microscopy analysis with antibodies against p53 (DO-7). This analysis revealed no nuclear translocation of p53 in HHV-6B-infected cells (Figure 3G).

### U19 Protein Inhibits p53-dependent Checkpoint Arrest

**Figure 4:**
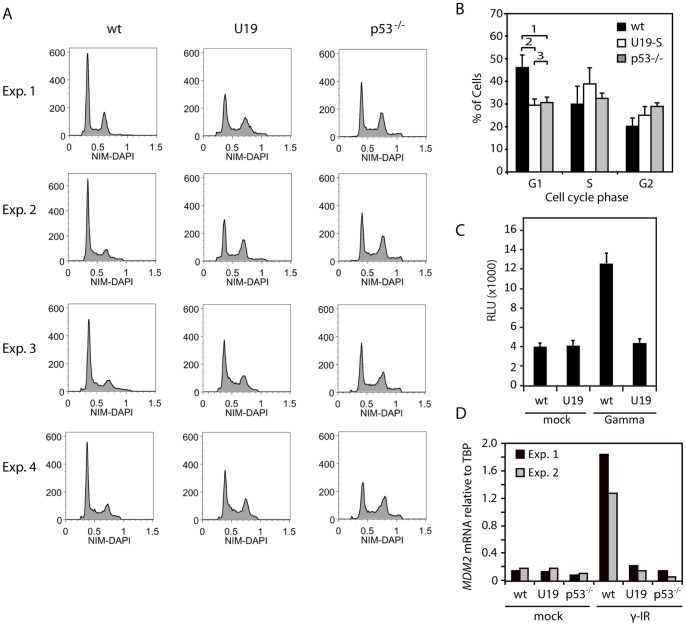
U19 expression inhibits p53-dependent check-point arrest. (**A**) Cell cycle analyses of wt HCT116 cells, HCT116 cells stably expressing U19, and HCT116-p53^−/−^ cells. The cells were stained with NIM-DAPI and analyzed by flow cytometry. Four independent experiments are shown. (**B**) Quantification of the G1, S and G2 distributions, using the Dean-Jett-Fox algorithm in the Flow-Jo software program. An average of the four independent experiments from (A) is shown. Error bars indicate SD. The p values in G1 are: 1) p = 0.0005; 2) p = 0.0003; and 3) p = 0.49 (p values were determined using a t-test). (**C**) Analysis of p21 promoter activation using a p21-pro-*luciferase* construct. HCT116 wt cells and HCT116 cells stably expressing U19 were transfected with a p21-Luc promoter construct, γ-irradiated (30 Gy followed by 24 hrs of incubation) and analyzed for luciferase induction. Y-axis depicts relative light units (RLU). A representative out of two experiments is shown. Values are average of triplicate measurements with error bars indicating SD. (**D**) Real-time PCR on mRNA from HCT116 wt, U19S and p53^−/−^ cells with *MDM2* or *TBP* specific primers. *MDM2* mRNA levels are represented relative to *TBP*. Measurements were performed in duplicate. Two independent experiments is shown.

We have previously demonstrated that HHV-6B transcribes mRNA from the *U19* ORF and that a FLAG-tagged U19 protein can be produced in trans [39]. We had noticed that the protein product from the *U19* ORF induced a cell cycle profile with reduced G1-arrest. To confirm that U19 could induce an impaired G1-checkpoint arrest and a p53-deficient-like cell cycle profile, we performed cell cycle analyses of the stable U19-expressing cell line, HCT116-U19-S [39], using a nuclear isolation method with DAPI stain (NIM-DAPI) flow cytometry and compared with the profiles obtained from HCT116-p53^−/−^ cells. As expected, HCT116-p53^−/−^ cells demonstrated a drop in the proportion of cells in G1, and an increase in the S and G2 population (Figures 4A and 4B), probably caused by a lack of p53-induced checkpoint arrest in G1/S and G2/M [40]. Analysis of the U19-S cells yielded similar results with a drop in G1 and an increase in S and G2, indicating a dysfunctional checkpoint arrest (Figures 4A and 4B). These data indicated that cells expressing U19, despite their elevated levels of p53, behaved similar to cells lacking p53.

P53-dependent checkpoint arrest is conveyed through p21, which inhibits cyclin-dependent kinase 2 (CDK2). CDK2 is an inhibitor of Rb, which acts as an inhibitor of the transcription factor E2F-1. E2F-1 is needed for progression into the S-phase of the cell cycle. If U19 inhibits p53 activity during normal cell cycle, no p21 transcription would be expected in these cells. We examined the ability of HCT116-U19-S cells to transactivate a p21 promoter-driven *luciferase* gene. Expression of the WWP-*luc* plasmid containing the wt WAF1 (p21) gene in HCT116 wt cells led to a three-fold increase in luciferase activity after γ irradiation when compared with untreated cells (Figure 4C). When WWP-*luc* was expressed in HCT116-U19-S cells, no induction of luciferase activity after γ irradiation was detectable (Figure 4C). This finding suggests that U19-expressing cells are deficient in normal p53-induced checkpoint arrest.

To further address p53 target genes, we conducted real-time PCR with *MDM2* specific primers on mRNA from wt cells, U19S cells or p53^−/−^ cells either mock-treated cells or cells treated with γ radiation. Similarly to our observations with the WWP-*luc* assay, we found γ-induction of *MDM2* in wt cells but not in U19S or p53^−/−^ cells (Figure 4D).

### HHV-6B Protein U19 Rescues Cells from p53-dependent Cell Death

**Figure 5:**
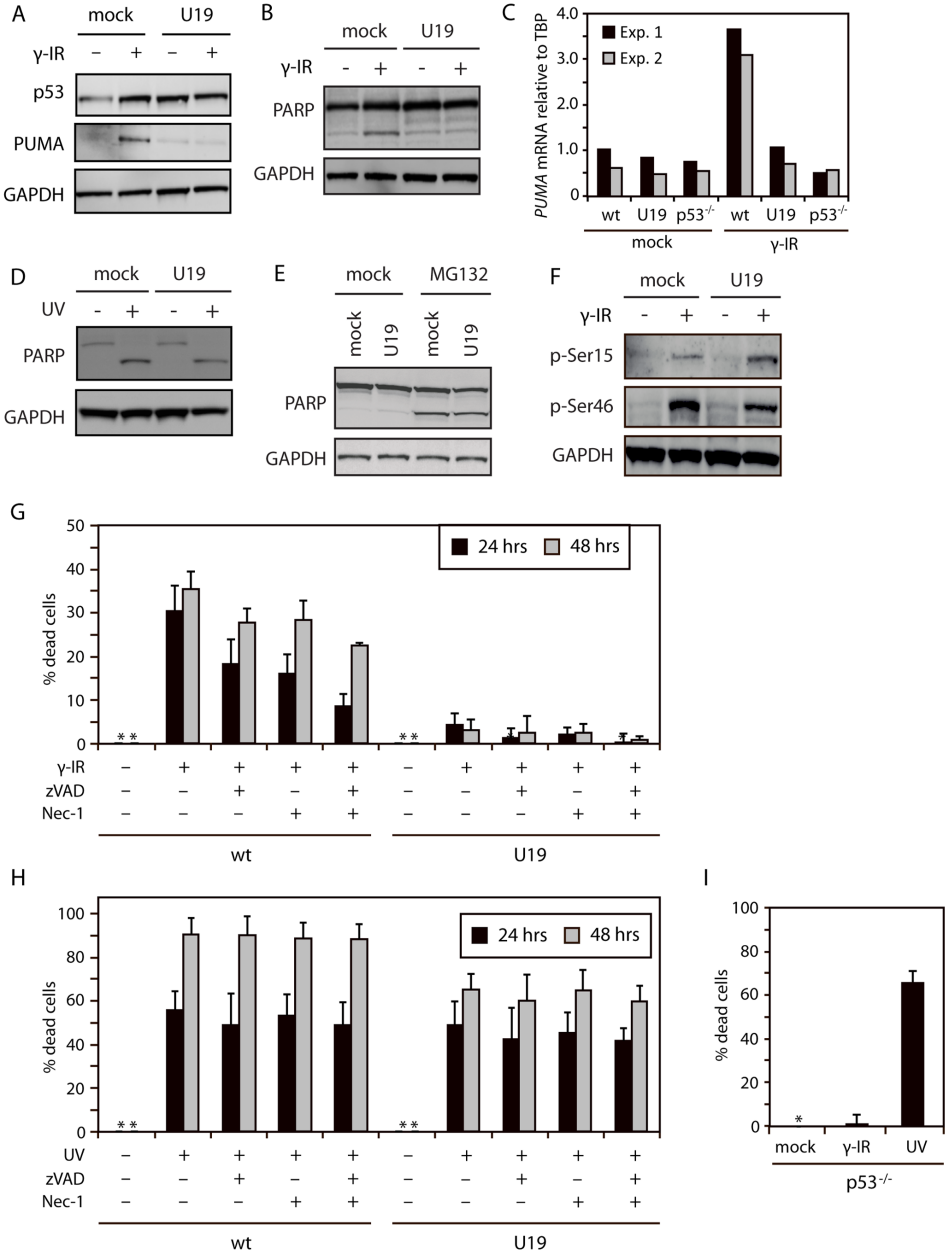
U19 inhibits p53-dependent apoptosis. (**A**) Western blot analyses with antibodies against p53, PUMA, and GAPDH on lysates from HCT116 cells transduced with an empty or a U19 lentivirus vector and treated with or without γ radiation (30 Gy) followed by incubation for 24 hrs. (**B**) Western blot analyses with antibodies against PARP and GAPDH on lysates from HCT116 cells transduced with an empty or a U19 lentivirus vector and treated with or without γ radiation (30 Gy) followed by incubation for 24 hrs. (**C**) Real-time PCR on mRNA from HCT116 wt, U19S and p53^−/−^ cells with *PUMA* or *TBP* specific primers. *PUMA* mRNA levels are represented relative to *TBP*. Measurements were performed in duplicate. Two independent experiments is shown. (**D**) Western blot analyses with antibodies against PARP and GAPDH on lysates from HCT116 cells transduced with an empty or a U19 lentivirus vector and treated with or without UV radiation (60 J/cm2) followed by 4 hrs incubation. (**E**) Western blot analyses with antibodies against PARP and GAPDH on lysates from HCT116 cells transduced with an empty or a U19 lentivirus vector and treated with or without MG132 for 24 hrs. (**F**) Western blot analyses with antibodies against p53 p-Ser15, p53 p-Ser46, and GAPDH on lysates from HCT116 cells transduced with an empty or a U19 lentivirus vector and treated with or without γ radiation (30 Gy) followed by 24 hrs of incubation. (**G & H**) HCT116 mock-LV (wt) and U19-LV (U19) cells where treated with or without zVAD, nec-1 or both for 1 hr followed by γ irradiation (30 Gy) or UV treatment (60 J/cm^2^) for 24 or 48 hrs. Cell viability was assessed by measuring intracellular ATP-levels. Data are shown as % dead cells calculated relative to untreated cells. Measurements were performed in duplicate. An average of three independent experiments is shown. Error bars represent SD. (**I**) HCT116 p53^−/−^ cells were treated with or without γ radiation (30 Gy) or UV (60 J/cm^2^) for 24 hrs followed by analysis of intracellular ATP levels. Measurements were performed in duplicate. An average of three independent experiments is shown. Error bars represent SD.

To determine if p53 in U19-expressing cells were broadly functionally inactive, lentiviral-transduced U19 cells (U19-LV) were γ-irradiated and analyzed by Western blotting with antibodies against p53, PUMA and PARP. As expected, mock-LV cells showed increased levels of p53 after γ irradiation. This correlated with an increase in expression of PUMA and an increase in cleavage of PARP, indicating induction of apoptosis. In contrast, U19-LV cells had increased levels of p53, and did not upregulate PUMA or cleave PARP upon γ radiation (Figures 5A and 5B). Moreover, when wt, U19S or p53^−/−^ cells were analyzed by real-time PCR for *PUMA* mRNA induction after γ irradiation, only wt cells responded with increased levels of *PUMA* mRNA (Figure 5C).

To determine if U19-expressing cells could still die through p53-independent death pathways, U19-LV and mock-LV cells were treated with UV radiation and MG132. Similarly to observations with HHV-6B-infected cells, U19-expressing cells died from these treatments (Figures 5D and 5E). To examine whether the inhibition of p53 activity was due to reduced post-translational phosphorylations, mock-LV and U19-LV cells were γ-irradiated and cell lysates were subsequently analyzed by Western blotting with antibodies against p53 pSer15 and pSer46. Both mock and U19 cells showed increased phosphorylation on both Ser15 and Ser46 following γ irradiation (Figure 5F). This demonstrated that although p53 is phosphorylated on Ser46, it does not mediate cell death in the presence of the HHV-6B U19 protein.

Recently, new types of programmed cell death have been described, most prominently the non-apoptotic, necrotic-like cell death termed necroptosis [41]. This type of cell death appears to act as a back-up mechanism when apoptosis fails, such as during herpesvirus-infections, where caspase-8 is often blocked by viral proteins [42], [43]. Necroptosis can be induced by inhibition of apoptosis, using the pan-caspase inhibitor zVAD-fmk. Conversely, necroptosis is dependent on RIP1 kinase activity, which can by inhibited by necrostatin-1 (nec-1).

In order to determine whether U19 expressing cells where capable of dying through necroptosis following γ radiation, wt and U19-LV cells where treated with or without zVAD or nec-1 followed by γ irradiation and subsequent incubation for 24 or 48 hrs. The cells were analyzed for intracellular ATP levels as an indirect measurement of cell death. Alone, zVAD or nec-1 induced a minor reduction in cell death (Figure 5G), which was specifically pronounced after 24 hrs treatment when the inhibitors were added together (Figure 5G). In agreement with analysis by WB (Figure 5A and B), U19-LV cells did not show any signs of cell death after γ irradiation, and this was not changed by the presence of zVAD (Figure 5G).

To determine if the inability of U19 to block UV-induced cell death was due to a switch to necroptosis, we treated wt and U19-LV cells with or without zVAD or nec-1 followed by UV treatment and incubation for 24 or 48 hrs. Cell death was measured by ATP-analysis. Both wt and U19-LV cells died from UV treatment (Figure 5H). Surprisingly there was little or no effect from treating the cells with zVAD, nec-1 or zVAD/nec-1. However, the U19-LV cells did appear to die with slightly delayed kinetics when compared with wt cells.

In order to verify that UV radiation killed cells in a p53-independent manner and γ radiation killed the cells in a p53-dependent manner, we treated HCT116 p53^−/−^ cells with or without UV radiation or γ radiation for 24 hrs and analyzed the cells by ATP-assay. As expected, p53^−/−^ cells died after UV treatment, but were unaffected after γ irradiation (Figure 5I).

## Discussion

The tumor suppressor protein p53 is an important cellular antiviral factor that may induce apoptosis in infected cells, if not blocked by viral or cellular proteins. Many viruses have therefore evolved mechanisms to evade the response from p53 [44]. Since HHV-6B-infected cells do not die despite elevated levels of p53, we speculated that p53 might be inactivated during infection. To address this question, we performed both mRNA and protein analysis during cell death induced by p53-dependent and -independent pathways. These experiments demonstrated that HHV-6B infection rescued the cells from p53-dependent, but not p53-independent cell death.

Takemoto et al. [26] have previously demonstrated that infection of MOLT3 cells by the HST strain rescued the cells from UV radiation-induced cell death. Our results showed that infection of both permissive MOLT3 cells and non-permissive HCT116 cells by the PL1 strain failed to protect cells against UV radiation-induced apoptosis. Infection did reduce the rate of cell death at low-level UV-treatment, but death was still correlated to the amount of UV-treatment similar to untreated cells. The discrepancy between the previous study and our study remains unexplained, but might be caused by the use of different strains of virus. Alternatively, the type of UV radiation might have been different. Either way, this is an interesting observation that requires further investigation with comparison of different strains of virus.

Most of the functional activities of p53 occur in the nucleus, where p53 acts as a transcriptional activator of genes involved in numerous processes, including cell cycle arrest, pro-inflammation and cell death. Although p53 is primarily found in the cytoplasm during infection, we showed that p53 pSer46 is almost exclusively found in the nucleus. Sequestration of p53 in the cytoplasm may therefore not alone explain the apparent lack of p53 effector functions. Ser15 phosphorylation is primarily involved in stabilization of p53 and induction of cell cycle arrest, whereas additional phosphorylation on Ser46 is important for cells to undergo apoptosis [38], [45]. We found that Ser46 was markedly phosphorylated similar to that observed for other p53-Ser residues examined [24], [36]. This demonstrated that nuclear p53 contains pro-apoptotic modifications, yet the cells did not die. The reasons for this remain to be defined.

We were able to demonstrate that U19 expression led to a cell cycle profile resembling that of p53 knock-out cells. This is in agreement with our findings that U19-expressing cells fail to transcribe p21. The observed cell cycle profile in U19-expressing cells is not similar to the profile obtained from HHV-6B-infected cells [33]. This may not be surprising as HHV-6B-infected cells have been shown to arrest in G1 independently of p53 [24], [33]. Other viral proteins must thus be responsible for the induction of G1 arrest during the infection. Furthermore, we found that U19 expression induced stabilization and functional inactivation of p53. In contrast to wt cells, but similar to HHV-6B-infected cells, the U19-expressing cells did not upregulate PUMA expression upon γ irradiation nor did they die or arrest from the accumulated high level of p53. Nevertheless, cells expressing U19 were still able to die from p53-independent apoptosis. Switching the cells to the necroptotic pathway by the use of zVAD did not sensitize U19-expressing cells to γ-induced cell death. This indicates that after γ irradiation both the apoptotic and necroptotic pathways are dependent on p53-signaling. UV-induced cell death was inhibited by neither zVAD nor nec-1. Surprisingly co-treatment with zVAD and nec-1 also failed to rescue the cells from UV-induced cell death. UV radiation may thus induce death through another pathway than apoptosis or necroptosis.

In conclusion, U19 plays an important role in the rescue of p53-induced cell death during HHV-6B infection. Despite this, U19 may not be solely responsible for p53 inactivation during HHV-6B infection. Since *U19* is an early gene, HHV-6B may encode an immediate early inhibitor of p53, or include a p53 inhibitory protein in the virion, thereby carrying it into newly infected cells. The latter hypothesis is supported by the finding that the p53-interacting protein U14, is present in the virion [46]. Alternatively, the virus may instead need the activities of p53 at an early stage during infection. This is known from other viruses, including HCMV [22], [23].

## Supporting Information

Table S1 Genes used for heatmap analysis. Raw data from the array analysis are shown.
(DOCX)
