# Supplementary material for: Inhibition of p53-Dependent, but Not p53-Independent, Cell Death by U19 Protein from Human Herpesvirus 6B
Source: PLoS One. 2013 Mar 26;8(3):e59223. doi: 10.1371/journal.pone.0059223 (PMC3608612; doi:10.1371/journal.pone.0059223)
Supplement: Table S1 — Genes used for heatmap analysis. Raw data from the array analysis are shown. (DOCX) [file pone.0059223.s001.docx]

Table S1. Genes used for heatmap analysis.

| Unigene(Avadis) | Gene Symbol | wt_signal | wt_g_signal | wt_6B_signal | wt_6B_g_signal | p53-/-_signal | p53-/-_g_signal | wt_vs_wt_g_Signal Log Ratio | wt_vs_wt_6B_Signal Log Ratio | wt_vs_p53-/-_Signal Log Ratio | wt_g_vs_p53-/-_g_Signal Log Ratio | wt_g_vs_wt_6B_g_Signal Log Ratio | wt_6B_vs_wt_6B_g_Signal Log Ratio | wt_6B_vs_p53-/-_Signal Log Ratio |
| --- | --- | --- | --- | --- | --- | --- | --- | --- | --- | --- | --- | --- | --- | --- |
| Hs.137569 | TP73L | 2,2 | 15,5 | 10,9 | 18,1 | 3,8 | 10,2 | -3 | -2,4 | -0,6 | 0,5 | -0,6 | -1,2 | 1,5 |
| Hs.349094 | SH2D1A | 5,8 | 32,3 | 30,2 | 2,3 | 35 | 11 | -2,5 | -2,6 | -2,5 | 1,3 | 3,6 | 4 | 0,2 |
| Hs.5353 | CASP10 | 4 | 20,8 | 26 | 27,4 | 39,8 | 55,2 | -2,2 | -2,2 | -3,3 | -1,3 | -0,6 | -0,3 | -0,7 |
| Hs.616962 | GDF15 | 718,5 | 2764,9 | 538 | 1550,2 | 69,4 | 109,7 | -2 | 0,4 | 3,4 | 5 | 0,8 | -1,5 | 3 |
| Hs.82222 | SEMA3B | 11,8 | 33,3 | 32,5 | 20,5 | 21,5 | 16,9 | -2 | -1,4 | -0,4 | 0,9 | 0,2 | 0,8 | 0,8 |
| Hs.492261 | TP53INP1 | 6,1 | 27,9 | 3,8 | 5,8 | 6,2 | 4,3 | -2 | 0,1 | -0,1 | 2,3 | 2 | -0,2 | -0,3 |
| Hs.567303 | MDM2 | 2,7 | 15,3 | 3,2 | 13 | 12,6 | 15,2 | -1,9 | -1,2 | -2,1 | 0,5 | 0,2 | -1,9 | -1,7 |
| Hs.537002 |  | 0,7 | 2,9 | 6,5 | 2,3 | 0,9 | 3,4 | -1,9 | -2,6 | 0,4 | -0,4 | 0,7 | 0,8 | 1,3 |
| Hs.519162 | BTG2 | 158 | 522,9 | 128,3 | 282,2 | 123 | 143,6 | -1,8 | 0,1 | 0,6 | 2,3 | 0,8 | -1,1 | 0,5 |
| Hs.244139 | FAS | 120,7 | 366,2 | 95,8 | 223,3 | 54,1 | 48,7 | -1,7 | 0,2 | 0,8 | 2,9 | 0,6 | -1 | 0,9 |
| Hs.450230 | IGFBP3 | 1,9 | 8,2 | 2,3 | 3,8 | 2,9 | 3,3 | -1,7 | -0,4 | -0,5 | 0,9 | 0,8 | 0,1 | -0,1 |
| Hs.436023 | PRDM1 | 2,3 | 7,7 | 7,2 | 9,1 | 82,2 | 69,4 | -1,7 | -1,9 | -4 | -2,8 | -0,2 | 0,1 | -3,2 |
| Hs.492261 | TP53INP1 | 153,2 | 598,1 | 104,4 | 319,1 | 38,6 | 59,8 | -1,7 | 0,4 | 1,9 | 2,9 | 0,8 | -1,2 | 1,2 |
| Hs.210343 | BCL2L14 | 0,9 | 7,6 | 9,4 | 4,2 | 23,8 | 20,2 | -1,6 | -3,2 | -3,8 | -1,2 | 1 | 1,8 | -1 |
| Hs.370771 | CDKN1A | 956,3 | 2911,4 | 737,7 | 1764,4 | 365,9 | 562,9 | -1,6 | 0,3 | 1,4 | 2,4 | 0,7 | -1,2 | 1,1 |
| Hs.526464 | PML | 4,4 | 15,8 | 28,3 | 31 | 6,5 | 3,3 | -1,6 | -2,7 | -0,4 | 2 | -1 | 0,2 | 2,9 |
| Hs.4865 | SCN3B | 2,3 | 8,6 | 5,1 | 5,2 | 5 | 20,2 | -1,6 | -0,4 | -0,3 | -1,5 | 0,7 | 0,1 | -0,3 |
| Hs.244139 | FAS | 156,9 | 428,4 | 125,9 | 294,9 | 34,4 | 38,8 | -1,5 | 0,2 | 2,3 | 3,2 | 0,7 | -1 | 1,8 |
| Hs.244139 | FAS | 91,2 | 265,4 | 98,8 | 162,8 | 32,1 | 31 | -1,5 | -0,1 | 1,6 | 3,3 | 0,6 | -0,8 | 1,8 |
| Hs.460 | ATF3 | 301,5 | 750,7 | 309,1 | 488,3 | 180,4 | 234,1 | -1,4 | 0 | 0,8 | 1,8 | 0,7 | -0,8 | 0,8 |
| Hs.244139 | FAS | 238,5 | 658,6 | 234,6 | 416,9 | 65,2 | 86,3 | -1,4 | 0,1 | 1,8 | 3,2 | 0,7 | -0,9 | 1,8 |
| Hs.137569 | TP73L | 3,8 | 3,1 | 20,2 | 10,2 | 2,8 | 4,2 | -1,4 | -2,2 | -0,5 | 0,5 | -0,1 | 0,9 | 2,6 |
| Hs.591336 | SESN1 | 159,6 | 430,1 | 145,4 | 189,4 | 77,1 | 83,2 | -1,3 | 0,1 | 0,9 | 2,2 | 0,8 | -0,7 | 0,7 |
| Hs.460 | ATF3 | 20,3 | 48,6 | 27,1 | 33,3 | 30,8 | 32,1 | -1,2 | -0,9 | -1,6 | 0,8 | 0,4 | -0,1 | -0,5 |
| Hs.467020 | BBC3 | 54,6 | 139,1 | 74,5 | 116,1 | 62,6 | 85,9 | -1,2 | 0 | 0,7 | 0,6 | 0,5 | -0,4 | 0,5 |
| Hs.1407 | EDN2 | 6,1 | 12,8 | 17,7 | 4,9 | 6,4 | 3,3 | -1,2 | -1,3 | -0,1 | 1,6 | 1,4 | 1,3 | 0,9 |
| Hs.69745 | FDXR | 330,3 | 705,3 | 297,8 | 504 | 167,3 | 200,5 | -1,1 | 0,2 | 1 | 1,8 | 0,5 | -0,7 | 0,8 |
| Hs.158932 | APC | 4,6 | 19,2 | 19,8 | 22,1 | 40,7 | 42,7 | -1 | -1,7 | -2,7 | -1,6 | -0,5 | -0,3 | -1,1 |
| Hs.210343 | BCL2L14 | 6,3 | 12 | 13,5 | 4,9 | 15,7 | 19 | -1 | -0,9 | -1,1 | 0,1 | 1,5 | 1,8 | -0,1 |
| Hs.80409 | GADD45A | 1355,3 | 2116,5 | 972,8 | 1441,1 | 901,9 | 1189 | -1 | 0,1 | 0,2 | 0,8 | 0,6 | -0,5 | 0 |
| Hs.396530 | HGF | 3,3 | 7 | 7 | 1,7 | 2,6 | 6 | -1 | -0,1 | 0,9 | 0,7 | 2,6 | 1,8 | 0,2 |
| Hs.450230 | IGFBP3 | 2,7 | 5,6 | 6,4 | 3 | 5,9 | 16,6 | -1 | -1 | -1,2 | -1,6 | 1 | 0,8 | -0,2 |
| Hs.567303 | MDM2 | 104,1 | 193,8 | 107,5 | 126,5 | 78,9 | 86 | -1 | 0 | 0,2 | 1,7 | 0,5 | -0,4 | 0,3 |
| Hs.460 | ATF3 | 122,9 | 154,1 | 86,2 | 122,5 | 81,3 | 72,8 | -0,9 | 0,1 | 0,3 | 1,5 | 0,5 | -0,3 | 0,3 |
| Hs.519162 | BTG2 | 54 | 182,5 | 45,7 | 103,4 | 22,7 | 22,8 | -0,9 | 0,1 | 1 | 1,7 | 0,5 | -0,8 | 0,5 |
| Hs.494529 | FANCC | 3,6 | 10,6 | 13,5 | 2,5 | 22,5 | 1,9 | -0,9 | -1,6 | -2,2 | 2 | 2,1 | 3,7 | -0,5 |
| Hs.398157 | PLK2 | 929,6 | 1770,1 | 992,1 | 1454,4 | 891,7 | 889,4 | -0,9 | -0,1 | 0,1 | 1 | 0,3 | -0,5 | 0,2 |
| Hs.632415 | PLK3 | 113,7 | 180,5 | 87,3 | 157,9 | 90 | 165,6 | -0,9 | 0,4 | 0,4 | 0,8 | 0,6 | -0,8 | 0 |
| Hs.552567 | APAF1 | 40,4 | 67,1 | 42,4 | 57,6 | 107,6 | 76,5 | -0,8 | -0,2 | -1,3 | -0,2 | 0,5 | -0,1 | -1,2 |
| Hs.651197 | DDB2 | 396,5 | 668,1 | 347 | 545,2 | 205,6 | 198,7 | -0,8 | 0,2 | 1,4 | 1,8 | 0,4 | -0,5 | 1,3 |
| Hs.523012 | DDIT4 | 354,8 | 849 | 588 | 733,6 | 435,5 | 384,4 | -0,8 | -0,3 | -0,1 | 0,7 | 0 | -0,5 | 0,1 |
| Hs.500466 | PTEN | 37,4 | 58,8 | 54,1 | 46,9 | 59,3 | 40,3 | -0,8 | -0,6 | -1 | 0,6 | 0,4 | 0 | -0,2 |
| Hs.55279 | SERPINB5 | 171,8 | 314,4 | 204,5 | 242,5 | 202,8 | 258,6 | -0,8 | 0 | -0,1 | 0,7 | 0,4 | -0,5 | -0,3 |
| Hs.521456 | TNFRSF10B | 910,4 | 1634,5 | 871,1 | 1147,4 | 872,6 | 811,2 | -0,8 | 0,1 | 0,1 | 0,9 | 0,4 | -0,4 | 0 |
| Hs.213467 | TNFRSF10D | 47,6 | 99,2 | 98,2 | 119,4 | 133 | 140,9 | -0,8 | -0,5 | -1 | -0,3 | -0,1 | -0,2 | -0,4 |
| Hs.96 | PMAIP1 | 765,1 | 1296,3 | 976,9 | 1149 | 1191,5 | 1159,2 | -0,8 | -0,3 | -0,6 | 0,1 | 0,2 | -0,2 | -0,3 |
| Hs.525572 | BDKRB2 | 8,6 | 19,3 | 4 | 7 | 6,4 | 2,2 | -0,7 | 1,8 | 1,5 | 2,2 | 1,6 | -1 | 0 |
| Hs.396530 | HGF | 0,3 | 1,8 | 0,4 | 6,1 | 5,4 | 1,1 | -0,7 | 0,6 | -3,2 | 0,4 | -2,1 | -3,7 | -3,9 |
| Hs.567303 | MDM2 | 79,8 | 124 | 63 | 75,4 | 45,9 | 56,6 | -0,7 | 0,2 | 0,5 | 1 | 0,4 | -0,6 | 0,3 |
| Hs.55279 | SERPINB5 | 12,1 | 20,2 | 8,4 | 24,6 | 5,8 | 17,8 | -0,7 | 0,9 | 0,7 | 0,2 | -0,2 | -1,8 | 0,2 |
| Hs.414795 | SERPINE1 | 12,3 | 20,2 | 16,1 | 19,6 | 13,8 | 17,7 | -0,7 | -0,2 | -0,1 | 0,1 | -0,1 | -0,3 | 0 |
| Hs.523718 | SFN | 253,1 | 463,5 | 201,6 | 300,8 | 981,5 | 1378,7 | -0,7 | 0,5 | -1,8 | -1,5 | 0,8 | -0,4 | -2,2 |
| Hs.213467 | TNFRSF10D | 386,1 | 625,7 | 591 | 766,6 | 853,6 | 861,3 | -0,7 | -0,7 | -1,2 | -0,4 | -0,2 | -0,3 | -0,4 |
| Hs.414795 | SERPINE1 | 53,2 | 95,3 | 58,3 | 78,8 | 174 | 165,7 | -0,6 | 0,4 | -1,7 | -0,8 | 0,7 | -0,4 | -1,8 |
| Hs.523718 | SFN | 2644,1 | 3975,5 | 1799,9 | 2434,3 | 6171,3 | 8131,7 | -0,6 | 0,5 | -1,3 | -1 | 0,7 | -0,3 | -1,8 |
| Hs.591834 | TNFRSF10A | 488,6 | 692,9 | 500,9 | 605 | 655,8 | 786,6 | -0,6 | -0,1 | -0,4 | 0 | 0,2 | -0,2 | -0,3 |
| Hs.69499 | TRIAP1 | 1380,4 | 2052,8 | 1133,9 | 1290,8 | 1262,3 | 1361,8 | -0,6 | 0,3 | 0,2 | 0,5 | 0,7 | -0,2 | -0,1 |
| Hs.501778 | TRIM22 | 10,6 | 14 | 9 | 10,3 | 1,9 | 0,4 | -0,6 | -0,1 | 2,3 | 4,9 | 0,8 | 0,2 | 2,7 |
| Hs.631546 | BAX | 51,4 | 79,3 | 46,5 | 70,2 | 73,2 | 88 | -0,5 | -0,2 | -0,4 | 0,2 | 0,1 | -0,3 | -0,2 |
| Hs.5353 | CASP10 | 13,5 | 23,6 | 26,1 | 12,2 | 28 | 29 | -0,5 | -0,8 | -0,8 | -0,6 | 0,4 | 0,5 | -0,2 |
| Hs.527778 | CD82 | 14,5 | 23,3 | 38,3 | 46,9 | 45,5 | 59,5 | -0,5 | -1,5 | -1,1 | -1,3 | -1,3 | -0,3 | -0,3 |
| Hs.518450 | HD | 40,3 | 83,5 | 58,2 | 67,1 | 95,8 | 54,4 | -0,5 | -0,4 | -0,8 | 0,3 | -0,1 | -0,4 | -0,4 |
| Hs.592290 | LRDD | 144,1 | 152,9 | 138,2 | 181,4 | 139 | 107,8 | -0,5 | 0,1 | -0,2 | 0,2 | 0,1 | -0,5 | 0 |
| Hs.113275 | P2RXL1 | 18,4 | 24,5 | 5 | 32,1 | 56,8 | 25,2 | -0,5 | 2,1 | -1,3 | 0,1 | 0 | -2,4 | -3,7 |
| Hs.6061 | PRKAB1 | 301,8 | 360,7 | 269,6 | 319,8 | 215,4 | 237,8 | -0,5 | -0,1 | 0,4 | 0,7 | 0,1 | -0,2 | 0,4 |
| Hs.414795 | SERPINE1 | 95,7 | 170,3 | 74,2 | 100,6 | 249,5 | 325,6 | -0,5 | 0,7 | -1,3 | -1 | 0,8 | -0,4 | -2,2 |
| Hs.170009 | TGFA | 311,3 | 439,2 | 290,5 | 316 | 736,2 | 811,9 | -0,5 | -0,1 | -1,5 | -1,1 | 0,2 | -0,3 | -1,4 |
| Hs.170009 | TGFA | 40,4 | 72,5 | 54,5 | 49,2 | 92,1 | 112,1 | -0,5 | -0,1 | -0,8 | -0,6 | 0,2 | -0,3 | -0,9 |
| Hs.521456 | TNFRSF10B | 208,9 | 292,2 | 196 | 250,5 | 223,9 | 227 | -0,5 | 0 | 0,1 | 0,6 | 0,3 | -0,4 | 0 |
| Hs.521456 | TNFRSF10B | 205,6 | 321,2 | 194 | 220 | 204,5 | 189 | -0,5 | 0,2 | 0,2 | 0,8 | 0,5 | -0,2 | -0,2 |
| Hs.96 | PMAIP1 | 1607 | 2446,9 | 1676,3 | 1959,3 | 1963,7 | 2395,1 | -0,5 | -0,1 | -0,3 | -0,1 | 0,2 | -0,2 | -0,2 |
| Hs.504545 | C12orf5 | 566,8 | 817,5 | 532,1 | 626,2 | 526,9 | 621,5 | -0,4 | 0,1 | 0 | 0,3 | 0,3 | -0,2 | -0,1 |
| Hs.5353 | CASP10 | 27,8 | 30,1 | 34,7 | 10,7 | 10,3 | 9,9 | -0,4 | -0,4 | 1,4 | 1,3 | 1,6 | 1,7 | 1,6 |
| Hs.488293 | EGFR | 10,4 | 15,2 | 13,1 | 2,6 | 17,8 | 20,1 | -0,4 | -0,8 | -1 | -0,3 | 1,6 | 1,9 | 0 |
| Hs.2250 | LIF | 190,3 | 216,7 | 181,4 | 252,8 | 73,6 | 111 | -0,4 | -0,2 | 0,8 | 1,1 | -0,2 | -0,3 | 0,7 |
| Hs.592290 | LRDD | 80,2 | 87,6 | 91,8 | 82,4 | 68,2 | 84,9 | -0,4 | -0,2 | -0,2 | -0,1 | -0,1 | 0 | 0,1 |
| Hs.574741 | NLRC4 | 1,5 | 3,8 | 2,4 | 9,9 | 0,5 | 3,3 | -0,4 | -0,2 | 0,6 | -0,5 | -1,4 | -1,8 | 0,7 |
| Hs.444975 | PLAGL1 | 546,1 | 658,8 | 519,5 | 544,7 | 315,2 | 368,1 | -0,4 | -0,1 | 0,2 | 0,5 | 0 | -0,1 | 0,4 |
| Hs.444975 | PLAGL1 | 392,7 | 622,4 | 478,7 | 412,9 | 282,6 | 335,2 | -0,4 | -0,2 | 0,1 | 0,7 | 0,2 | -0,1 | 0,4 |
| Hs.436023 | PRDM1 | 7 | 9,3 | 11,8 | 9,5 | 53,5 | 12,6 | -0,4 | -0,2 | -3,4 | -0,2 | 0 | -0,2 | -3,3 |
| Hs.523718 | SFN | 2942,4 | 3907,3 | 1891,1 | 2451 | 5977,4 | 7584 | -0,4 | 0,6 | -1 | -1 | 0,7 | -0,3 | -1,6 |
| Hs.50649 | TP53I3 | 166,2 | 196,1 | 140,7 | 141,5 | 89,6 | 75,5 | -0,4 | 0,2 | 1 | 1,5 | 0,3 | -0,1 | 0,9 |
| Hs.210343 | BCL2L14 | 0,5 | 0,6 | 0,6 | 8,6 | 1 | 1,1 | -0,3 | 0 | -0,2 | -1,2 | -3,6 | -3,4 | 0,3 |
| Hs.2490 | CASP1 | 29,2 | 29,6 | 45,9 | 32,5 | 79,4 | 39,5 | -0,3 | -0,6 | -0,6 | -0,1 | 0 | 0,4 | -0,1 |
| Hs.488293 | EGFR | 31,8 | 38,2 | 47,5 | 39 | 53,1 | 48,9 | -0,3 | -0,3 | -0,8 | -0,4 | -0,2 | -0,2 | -0,5 |
| Hs.494529 | FANCC | 9,1 | 12,5 | 17,7 | 19,9 | 15,3 | 23,2 | -0,3 | -0,5 | -0,6 | -0,8 | -0,5 | -0,4 | 0 |
| Hs.147433 | PCNA | 4371 | 5142,1 | 4204,6 | 5278 | 3736,7 | 4925,4 | -0,3 | 0,1 | 0,2 | 0,2 | 0 | -0,3 | 0,1 |
| Hs.6061 | PRKAB1 | 251,5 | 259,9 | 184,3 | 203,9 | 179,3 | 151 | -0,3 | 0,1 | 0,3 | 0,7 | 0,4 | -0,2 | 0,2 |
| Hs.395482 | PTK2 | 30,8 | 41,5 | 29 | 36,7 | 47,3 | 31,1 | -0,3 | 0,1 | -0,4 | 0,3 | 0 | -0,1 | -0,3 |
| Hs.377992 | RABGGTA | 81,5 | 86,4 | 77,8 | 65,9 | 199,6 | 163,2 | -0,3 | 0,1 | -1,4 | -1 | 0,2 | -0,2 | -1,2 |
| Hs.408528 | RB1 | 383,5 | 466 | 403,8 | 400,9 | 622,7 | 579,3 | -0,3 | 0 | -0,6 | -0,3 | 0,2 | 0 | -0,5 |
| Hs.512592 | RRM2B | 463,4 | 580,3 | 441,3 | 485,9 | 423,1 | 366 | -0,3 | 0 | 0,1 | 0,7 | 0,3 | -0,1 | 0,2 |
| Hs.591834 | TNFRSF10A | 332,9 | 346 | 289,4 | 356,4 | 368,8 | 361,6 | -0,3 | 0 | -0,2 | 0 | 0,2 | -0,2 | -0,2 |
| Hs.44532 | UBD | 1,4 | 3,6 | 1,1 | 1,5 | 3 | 2 | -0,3 | -0,4 | -0,6 | -0,1 | -0,1 | -0,2 | -0,3 |
| Hs.158932 | APC | 278,7 | 311,7 | 315,9 | 318,1 | 375,1 | 275,6 | -0,2 | -0,2 | -0,4 | 0,2 | 0 | 0,1 | -0,3 |
| Hs.500483 | ACTA2 | 102,6 | 132,4 | 92,2 | 81,9 | 47,6 | 62,9 | -0,2 | 0,3 | 0,6 | 0,3 | 0,5 | -0,1 | 0,1 |
| Hs.478588 | BCL6 | 5,7 | 3 | 4 | 11,3 | 3,3 | 2,4 | -0,2 | -0,2 | 0 | -0,3 | -1,7 | -1,3 | 0,1 |
| Hs.368282 | RP11-125A7.3 | 222,4 | 210 | 245,6 | 265,8 | 265,2 | 227,3 | -0,2 | 0 | -0,1 | 0,2 | 0 | 0 | 0 |
| Hs.2490 | CASP1 | 27,6 | 44,4 | 33 | 43,6 | 37,8 | 14,5 | -0,2 | 0,1 | 0 | 1,6 | -0,1 | -0,2 | -0,1 |
| Hs.5353 | CASP10 | 4 | 8,1 | 19,1 | 4,4 | 9,4 | 7,5 | -0,2 | -1,6 | -0,5 | -0,2 | 0 | 1,2 | 0,8 |
| Hs.79101 | CCNG1 | 2715,6 | 2931,3 | 2567,2 | 2877,5 | 2333,7 | 1955,9 | -0,2 | 0,1 | 0,3 | 0,7 | 0,1 | -0,2 | 0,2 |
| Hs.121575 | CTSD | 40,6 | 51,8 | 39,2 | 61,5 | 84,9 | 73,1 | -0,2 | -0,4 | -0,5 | -0,2 | -0,2 | -0,3 | -0,4 |
| Hs.586423 | EEF1A1 | 493,4 | 552,5 | 398,3 | 447,6 | 465,1 | 408,8 | -0,2 | 0,3 | 0,1 | 0,4 | 0,4 | 0 | -0,2 |
| Hs.488293 | EGFR | 294 | 329,6 | 271,3 | 285,1 | 442,5 | 450,5 | -0,2 | 0,1 | -0,6 | -0,6 | 0,1 | 0 | -0,7 |
| Hs.545196 | GML | 11,6 | 16,7 | 12,5 | 5 | 26,9 | 2,3 | -0,2 | 0,1 | -0,8 | 3,2 | 1,7 | 1,7 | -0,6 |
| Hs.72956 | HIC1 | 1,2 | 0,7 | 0,5 | 0,8 | 0,6 | 2 | -0,2 | 0,2 | -0,3 | -0,3 | 0,1 | -0,4 | -0,2 |
| Hs.531081 | LGALS3 | 25,6 | 29 | 27,4 | 16,6 | 14,3 | 18 | -0,2 | -0,5 | 0,5 | 0,2 | 0,3 | 0,6 | 0,6 |
| Hs.195364 | MLH1 | 233,8 | 251,6 | 257,6 | 281,6 | 174,9 | 243,9 | -0,2 | -0,3 | 0,4 | 0,1 | -0,2 | -0,2 | 0,6 |
| Hs.113275 | P2RXL1 | 1,6 | 2,7 | 2,6 | 2,8 | 4,9 | 1,9 | -0,2 | -0,1 | -0,2 | -0,1 | 0 | 0,4 | 0,2 |
| Hs.526464 | PML | 22,8 | 28 | 25,3 | 25,8 | 28,2 | 28,8 | -0,2 | -0,6 | -0,5 | 0 | -0,1 | 0,2 | 0 |
| Hs.500466 | PTEN | 169,4 | 211,2 | 178,5 | 157,2 | 167,9 | 171,3 | -0,2 | -0,1 | 0 | 0,3 | 0,2 | 0,1 | 0,1 |
| Hs.500466 | PTEN | 354,3 | 393,4 | 391,9 | 373,4 | 346,5 | 311,1 | -0,2 | -0,2 | 0 | 0,3 | 0,1 | 0,1 | 0,2 |
| Hs.500466 | PTEN | 688 | 668,7 | 598,9 | 740,7 | 614,4 | 505 | -0,2 | -0,1 | 0,1 | 0,6 | 0,1 | 0,1 | 0,3 |
| Hs.500466 | PTEN | 19,2 | 25,4 | 17,9 | 20,1 | 15,5 | 11,1 | -0,2 | -0,2 | 0,2 | 0,6 | -0,1 | 0,2 | 0,1 |
| Hs.108957 | RPS27L | 3817,5 | 4580,9 | 3303 | 3908,7 | 2194,9 | 2314 | -0,2 | 0,2 | 0,8 | 0,9 | 0,2 | -0,3 | 0,6 |
| Hs.516484 | S100A2 | 199 | 239,8 | 406,4 | 387,5 | 478,9 | 480,5 | -0,2 | -1,2 | -1,3 | -1,3 | -0,9 | -0,1 | -0,3 |
| Hs.158932 | APC | 26,3 | 29 | 37,8 | 27,5 | 19,9 | 35,8 | -0,1 | -0,7 | 0,4 | -0,3 | 0 | 0,3 | 0,7 |
| Hs.591054 | BID | 271,2 | 311,8 | 273,8 | 293,4 | 167,8 | 200,5 | -0,1 | 0 | 0,9 | 0,5 | 0,1 | 0,1 | 0,8 |
| Hs.368282 | RP11-125A7.3 | 157,9 | 135,1 | 118,4 | 177,8 | 128,6 | 151,1 | -0,1 | 0,4 | 0,2 | 0,5 | 0,2 | -0,2 | -0,2 |
| Hs.507866 | RGC32 | 44 | 55,6 | 69,2 | 77,5 | 29,4 | 24,4 | -0,1 | -0,2 | 0,7 | 0,9 | -0,3 | -0,1 | 1,1 |
| Hs.121575 | CTSD | 12,1 | 11,7 | 19,8 | 30,4 | 6,2 | 7,7 | -0,1 | -0,3 | 2,5 | 1,1 | -0,1 | -0,3 | 2,2 |
| Hs.488293 | EGFR | 2,7 | 3,6 | 3,2 | 3,9 | 4,1 | 8,1 | -0,1 | 0 | -0,8 | -0,5 | 0 | -0,1 | -0,7 |
| Hs.171596 | EPHA2 | 641,2 | 700,6 | 572 | 576,8 | 813,9 | 748,6 | -0,1 | 0,2 | -0,7 | -0,5 | 0,4 | 0 | -0,9 |
| Hs.76686 | GPX1 | 3065,6 | 3236,2 | 2890,5 | 2952,8 | 1949,4 | 2158,2 | -0,1 | 0,1 | 0,7 | 0,6 | 0,1 | 0 | 0,5 |
| Hs.521181 | IRF5 | 73 | 80,6 | 74,9 | 67,6 | 82 | 65,6 | -0,1 | 0,1 | -0,2 | 0 | 0,4 | 0,2 | -0,2 |
| Hs.597656 | MSH2 | 906,8 | 995 | 878,4 | 912 | 688,8 | 866,7 | -0,1 | 0,1 | 0,5 | 0,3 | 0,1 | -0,1 | 0,4 |
| Hs.20930 | PCBP4 | 195,2 | 192 | 192,5 | 229,6 | 225,4 | 235,9 | -0,1 | 0,1 | -0,2 | -0,1 | 0,2 | 0 | -0,3 |
| Hs.444975 | PLAGL1 | 569,5 | 605,8 | 512,1 | 569,7 | 456,2 | 496,1 | -0,1 | 0,2 | 0,3 | 0,3 | 0,1 | -0,1 | 0,1 |
| Hs.500466 | PTEN /// PTENP1 /// LOC731292 | 499,9 | 533 | 534,3 | 565,3 | 655,1 | 583,3 | -0,1 | 0 | -0,4 | -0,2 | -0,1 | 0 | -0,3 |
| Hs.408528 | RB1 | 57,8 | 68,8 | 44,6 | 24,3 | 71,5 | 86 | -0,1 | 0,6 | -0,6 | -0,1 | 1,5 | 1,2 | -1,2 |
| Hs.128856 | SCARA3 | 66,4 | 63,2 | 77,5 | 74,5 | 51,4 | 57,2 | -0,1 | 0,2 | 0,4 | 0 | -0,1 | -0,1 | 0,5 |
| Hs.4865 | SCN3B | 0,9 | 1,3 | 1,2 | 2,3 | 0,6 | 0,9 | -0,1 | -0,3 | -0,3 | -0,5 | 0,2 | 0,4 | 0 |
| Hs.349094 | SH2D1A | 10,6 | 10,3 | 4,8 | 6,1 | 5,8 | 31 | -0,1 | 1,1 | 1,3 | -1,6 | 0,8 | 0,1 | -0,3 |
| Hs.352018 | TAP1 | 175,5 | 194,9 | 186,6 | 207,9 | 175,8 | 169,5 | -0,1 | -0,1 | 0,2 | 0,3 | -0,1 | -0,1 | 0,2 |
| Hs.145269 | TNFRSF10C /// MGC31957 | 25 | 23,6 | 18,4 | 23,6 | 23,1 | 33,7 | -0,1 | 0,4 | 0,2 | -0,4 | -0,2 | -0,4 | -0,1 |
| Hs.408312 | TP53 | 107,2 | 135,8 | 139,1 | 143,8 | 32,1 | 39,1 | -0,1 | -0,4 | 1,7 | 1,4 | -0,4 | -0,1 | 2,1 |
| Hs.192132 | TP73 | 70,3 | 79,9 | 73,5 | 88,9 | 52,7 | 73 | -0,1 | -0,2 | 0,2 | 0,1 | -0,2 | -0,2 | 0,8 |
| Hs.90303 | TSC2 | 18,4 | 12,6 | 23 | 9,9 | 9,3 | 31,8 | -0,1 | -0,2 | 0 | -1,1 | 0,4 | 1,1 | 0,9 |
| Hs.270279 | TYRP1 | 15 | 10,4 | 6,5 | 2,4 | 8,8 | 12,2 | -0,1 | -0,1 | 0,3 | -0,1 | 1,3 | 1,3 | 0,1 |
| Hs.643801 | CSPG2 | 163,1 | 202,4 | 139,3 | 116,3 | 52,4 | 43,3 | -0,1 | 0,3 | 1,4 | 2 | 0,7 | 0,2 | 1,3 |
| Hs.533655 | AIFM2 | 369,2 | 391,8 | 322,8 | 280 | 236 | 319,9 | 0 | 0,1 | 0,5 | 0,4 | 0,3 | 0 | 0,4 |
| Hs.533655 | AIFM2 | 159,6 | 169,3 | 151,1 | 135,3 | 127,4 | 118,8 | 0 | 0,2 | 0,4 | 0,4 | 0,2 | 0,1 | 0,4 |
| Hs.158932 | APC | 101,8 | 83 | 99,7 | 90 | 110,7 | 81,4 | 0 | 0 | -0,3 | 0,1 | -0,1 | 0,1 | -0,2 |
| Hs.591054 | BID | 264,5 | 266,6 | 299,9 | 304,4 | 185,7 | 220,5 | 0 | -0,2 | 0,3 | 0,2 | -0,2 | 0 | 0,6 |
| Hs.474150 |  | 91,2 | 84,6 | 68,5 | 44,6 | 61,5 | 71,9 | 0 | 0,2 | 0,2 | 0 | 0,6 | 0,1 | -0,2 |
| Hs.131226 | BNIP3L | 354,8 | 347,7 | 405 | 404,8 | 1098,8 | 802,8 | 0 | -0,2 | -1,7 | -1,1 | -0,2 | 0 | -1,4 |
| Hs.2490 | CASP1 | 1,1 | 0,7 | 5,3 | 0,9 | 5,7 | 2 | 0 | -2,4 | -3,1 | -1,2 | -0,1 | 2,2 | -0,6 |
|  | CASP6 | 417,8 | 409,1 | 392,8 | 370,8 | 253,7 | 219,6 | 0 | 0,1 | 0,9 | 0,9 | 0,2 | 0,1 | 0,7 |
|  | CASP6 | 334,6 | 339,1 | 337,5 | 312,1 | 245,2 | 255 | 0 | -0,1 | 0,2 | 0,2 | 0 | 0,1 | 0,3 |
| Hs.510409 | CCNK | 816,7 | 819,9 | 827,6 | 822,8 | 844 | 898,2 | 0 | -0,1 | -0,1 | -0,1 | 0 | 0 | 0 |
| Hs.183861 | CHMP4C | 371,7 | 415,3 | 335 | 304,8 | 350,5 | 315,8 | 0 | 0,2 | 0,2 | 0,2 | 0,4 | 0,2 | 0 |
| Hs.84190 | SLC19A1 | 11,1 | 10,2 | 13,7 | 23,4 | 12,8 | 11,5 | 0 | 0 | -0,2 | -0,3 | -0,7 | -0,6 | -0,2 |
| Hs.517356 | COL18A1 | 154,6 | 131,6 | 155,7 | 177,5 | 183,9 | 149,8 | 0 | 0,1 | -0,3 | -0,2 | -0,2 | 0 | -0,3 |
| Hs.83114 | CRYZ | 1189,8 | 1244,8 | 1216,2 | 1243 | 1076,4 | 989,1 | 0 | 0,1 | 0,3 | 0,4 | 0,1 | 0 | 0,2 |
| Hs.121575 | CTSD | 8,5 | 8,3 | 9,7 | 5,8 | 6,1 | 9,7 | 0 | 0 | -0,3 | -0,3 | -0,1 | -0,1 | -0,3 |
| Hs.531668 | CX3CL1 | 3,2 | 3,3 | 3,1 | 2,1 | 2,1 | 3,4 | 0 | -0,1 | -0,5 | -0,3 | -0,3 | 0,1 | -0,4 |
| Hs.518760 | FIP1L1 | 659 | 668,8 | 745,6 | 722,5 | 557,2 | 577,3 | 0 | -0,1 | 0,4 | 0,2 | -0,1 | 0 | 0,4 |
| Hs.631988 | DDR1 | 596,6 | 579,4 | 594,7 | 630,9 | 611,3 | 705,5 | 0 | 0,1 | -0,2 | -0,3 | -0,2 | -0,1 | -0,2 |
| Hs.631988 | DDR1 | 551,9 | 578,5 | 585,3 | 566,7 | 810,3 | 716,2 | 0 | 0,1 | -0,4 | -0,3 | -0,1 | -0,1 | -0,4 |
| Hs.202672 | DNMT1 | 1876,2 | 2078,4 | 1883,2 | 1960,3 | 1658,5 | 1881,6 | 0 | 0,1 | 0,1 | 0 | 0 | -0,1 | 0 |
| Hs.2128 | DUSP5 | 823,4 | 862 | 792,2 | 876,6 | 1659,3 | 1639,8 | 0 | 0,1 | -0,9 | -0,9 | 0 | -0,2 | -1,1 |
| Hs.396530 | HGF | 0,9 | 2 | 1,9 | 0,9 | 1,6 | 2,3 | 0 | 0,3 | 0 | 0,2 | 0 | 0,5 | 0 |
| Hs.37003 | HRAS | 453,7 | 533,5 | 515,1 | 574,7 | 568,4 | 654,9 | 0 | 0,2 | -0,2 | -0,3 | -0,1 | -0,1 | -0,2 |
| Hs.180414 | HSPA8 | 10066,3 | 9904,8 | 9272,7 | 11562,5 | 10011,7 | 10308,4 | 0 | 0,1 | 0 | -0,1 | -0,2 | -0,3 | -0,1 |
| Hs.591785 | IER3 | 5493,6 | 6015,3 | 3423,4 | 3770,5 | 5044,6 | 4924,2 | 0 | 0,7 | 0,1 | 0,2 | 0,6 | -0,1 | -0,6 |
| Hs.73105 | PMS2 /// PMS2CL | 352,4 | 321,9 | 366,2 | 340,1 | 212,4 | 225,5 | 0 | 0,1 | 0,4 | 0,4 | 0 | 0,2 | 0,4 |
| Hs.500466 | PTEN | 440,4 | 431,3 | 465,1 | 452,5 | 535 | 467 | 0 | -0,1 | -0,3 | -0,2 | 0 | 0 | -0,2 |
| Hs.395482 | PTK2 | 1083,2 | 1014,6 | 1093,8 | 1206,8 | 1303,8 | 1345,1 | 0 | -0,1 | -0,3 | -0,2 | 0 | 0,1 | -0,2 |
| Hs.221847 | SLC38A2 | 4395,1 | 4521,5 | 3913,2 | 4355,7 | 4509,7 | 4355,8 | 0 | 0,2 | -0,1 | 0 | 0,1 | -0,2 | -0,3 |
| Hs.137569 | TP73L | 17,2 | 26,3 | 14,2 | 16,1 | 19,3 | 10,3 | 0 | 0,5 | -0,2 | 0,6 | 0,8 | 0,5 | -0,3 |
| Hs.137569 | TP73L | 2,2 | 2,6 | 7,9 | 23,3 | 1 | 12,6 | 0 | -1,4 | -0,3 | -2,5 | -2,7 | -1 | 1,8 |
| Hs.192132 | TP73 | 2 | 4,7 | 2,7 | 5,1 | 7,2 | 4 | 0 | -0,2 | 0 | -0,2 | -0,2 | -0,2 | -0,7 |
| Hs.643801 | CSPG2 | 129,6 | 141,2 | 97,3 | 72,9 | 19,4 | 33,9 | 0 | 0,6 | 2,6 | 2,2 | 0,7 | 0,3 | 2,1 |
| Hs.643801 | CSPG2 | 216,8 | 209,8 | 140 | 142,8 | 43,4 | 48,9 | 0 | 0,7 | 1,9 | 2 | 0,8 | 0,1 | 1,5 |
| Hs.643801 | CSPG2 | 452,3 | 460,2 | 286,8 | 285,2 | 111,7 | 98,6 | 0 | 0,6 | 1,8 | 2 | 0,6 | 0,1 | 1,4 |
| Hs.524368 | VDR | 160,5 | 167,3 | 148,6 | 178,7 | 96,2 | 146,5 | 0 | 0,2 | 0,6 | 0,2 | 0 | -0,2 | 0,7 |
| Hs.210343 | BCL2L14 | 4,7 | 2,6 | 1,7 | 2,5 | 5,1 | 6,4 | 0,1 | 0,7 | -0,2 | -1,7 | 0,3 | -0,2 | -0,5 |
| Hs.478588 | BCL6 | 132,7 | 96,8 | 182,5 | 177,4 | 81,9 | 78,5 | 0,1 | -0,4 | 0,6 | 0,5 | -0,6 | -0,1 | 1 |
| Hs.591054 | BID | 767,6 | 734,7 | 728,8 | 716,2 | 555,2 | 630,2 | 0,1 | 0 | 0,4 | 0,2 | -0,1 | 0 | 0,3 |
| Hs.645371 | BIRC5 | 69,1 | 61,8 | 63 | 77,9 | 89,6 | 87,6 | 0,1 | -0,2 | -0,3 | -0,5 | -0,1 | 0,2 | -0,5 |
| Hs.131226 | BNIP3L | 234,9 | 229,3 | 295,4 | 294,8 | 881,2 | 662,5 | 0,1 | -0,2 | -1,7 | -1,4 | -0,3 | -0,1 | -1,5 |
| Hs.631988 | DDR1 | 716,4 | 703,9 | 641 | 666,4 | 821,1 | 828,1 | 0,1 | 0,2 | -0,3 | -0,3 | 0 | 0 | -0,4 |
| Hs.488293 | EGFR | 173,3 | 182,7 | 186,3 | 154,6 | 244,8 | 216,2 | 0,1 | 0 | -0,3 | -0,1 | 0,2 | 0,2 | -0,4 |
| Hs.494529 | FANCC | 122,7 | 138,3 | 128,3 | 113,2 | 118,1 | 137,7 | 0,1 | -0,1 | 0,1 | -0,2 | -0,1 | -0,1 | 0 |
| Hs.180414 | HSPA8 | 9892,8 | 9395,8 | 9034,3 | 11896,7 | 9753,9 | 10169,1 | 0,1 | 0,2 | 0 | -0,2 | -0,2 | -0,3 | -0,1 |
| Hs.180414 | HSPA8 | 8790,7 | 8238,2 | 7417,7 | 8615,3 | 7424,8 | 8528,8 | 0,1 | 0,2 | 0,1 | 0 | -0,1 | -0,3 | -0,1 |
| Hs.180414 | HSPA8 | 10618,3 | 10332,4 | 9479,7 | 12775,5 | 11021,6 | 10771,9 | 0,1 | 0,2 | 0,1 | -0,1 | -0,2 | -0,3 | -0,1 |
| Hs.148741 | IBRDC2 | 43 | 41,1 | 42 | 47,4 | 26,2 | 16 | 0,1 | 0,2 | 0,6 | 0,6 | 0 | 0,1 | -0,1 |
| Hs.521181 | IRF5 | 67,9 | 61,8 | 68,2 | 73,7 | 90,5 | 74,2 | 0,1 | 0 | -0,2 | -0,2 | -0,2 | 0 | -0,1 |
| Hs.132966 | MET | 1816,1 | 1858,3 | 1110,1 | 1085,1 | 2261,4 | 2240,4 | 0,1 | 0,5 | -0,3 | -0,2 | 0,5 | 0 | -0,8 |
| Hs.132966 | MET | 447,4 | 358,4 | 232 | 258,3 | 541,2 | 379,7 | 0,1 | 0,6 | -0,3 | -0,1 | 0,5 | -0,1 | -0,8 |
| Hs.513617 | MMP2 | 17,7 | 31,5 | 29,6 | 29,6 | 42,2 | 40,5 | 0,1 | 0,1 | -0,3 | -0,4 | -0,1 | 0,2 | -0,6 |
| Hs.647092 | NOS3 | 1,6 | 0,6 | 2,5 | 2,8 | 2,6 | 2,5 | 0,1 | -0,2 | 0,3 | 0,2 | -0,2 | -0,4 | 0 |
| Hs.467701 | ODC1 | 7052 | 6802,7 | 6215,1 | 6565,9 | 5989,8 | 6837,5 | 0,1 | 0,3 | 0,2 | -0,1 | 0 | -0,2 | 0 |
| Hs.520421 | PERP | 1545,9 | 1447,3 | 1676,4 | 1942,7 | 1199,1 | 1189,8 | 0,1 | -0,2 | 0,4 | 0,4 | -0,5 | -0,1 | 0,7 |
| Hs.520421 | PERP | 4365,1 | 3960,1 | 4546,5 | 5019,1 | 3013,3 | 2867,1 | 0,1 | -0,1 | 0,6 | 0,4 | -0,3 | -0,1 | 0,6 |
| Hs.526464 | PML | 8 | 4 | 23,3 | 30 | 4,5 | 9,4 | 0,1 | -1,5 | 0,2 | -0,1 | -2,4 | 0,1 | 1,8 |
| Hs.534573 | PML /// LOC161527 | 43,6 | 37,2 | 48,5 | 25,9 | 7,2 | 11 | 0,1 | -0,1 | 1,6 | 1,2 | 0,5 | 0,8 | 1,3 |
| Hs.632637 | PMS2 | 198,2 | 245,7 | 254,6 | 234,5 | 249,9 | 253,2 | 0,1 | -0,2 | 0 | 0,1 | -0,3 | 0 | 0,2 |
| Hs.395482 | PTK2 | 566,6 | 461,8 | 612,9 | 589,5 | 738,6 | 626 | 0,1 | -0,1 | -0,4 | -0,3 | -0,1 | 0,1 | -0,3 |
| Hs.523744 | RFWD2 | 484 | 364,1 | 386,8 | 409,2 | 307,6 | 388,7 | 0,1 | 0,2 | 0,3 | 0,2 | 0 | 0 | 0,2 |
| Hs.128856 | SCARA3 | 65,1 | 60,3 | 69,3 | 67,2 | 64,6 | 75,8 | 0,1 | 0,1 | 0 | -0,1 | 0 | 0 | 0 |
| Hs.221847 | SLC38A2 | 3397,7 | 3229,9 | 2750,5 | 3155,1 | 3755,3 | 3530,3 | 0,1 | 0,3 | -0,1 | 0 | 0 | -0,2 | -0,4 |
| Hs.221847 | SLC38A2 | 3351,2 | 3099,8 | 2748,4 | 3025 | 3564,2 | 3265,8 | 0,1 | 0,3 | -0,1 | -0,1 | 0,1 | -0,1 | -0,4 |
| Hs.170009 | TGFA | 39,9 | 34,9 | 34,6 | 21,3 | 63,6 | 69,5 | 0,1 | 0,1 | -1 | -1,2 | 0,6 | 0,8 | -0,9 |
| Hs.408312 | TP53 | 262,7 | 239,9 | 305 | 379,8 | 60,5 | 58,9 | 0,1 | -0,3 | 2,1 | 2,3 | -0,6 | -0,2 | 2,5 |
| Hs.369759 | TRPM2 | 201 | 170,1 | 190,6 | 162,4 | 168,2 | 192,7 | 0,1 | -0,1 | 0,1 | -0,1 | -0,2 | 0 | 0,2 |
| Hs.90303 | TSC2 | 111,1 | 89,5 | 107,8 | 108,8 | 142 | 93,9 | 0,1 | -0,2 | -0,3 | -0,2 | -0,2 | 0 | -0,1 |
| Hs.643801 | CSPG2 | 447,4 | 432,8 | 284,3 | 264,7 | 114,9 | 103,7 | 0,1 | 0,7 | 1,7 | 2 | 0,9 | 0,2 | 1,1 |
| Hs.501296 | ARID3A | 59,3 | 53,4 | 76,8 | 69,9 | 88,9 | 67,6 | 0,2 | -0,2 | -0,5 | -0,4 | -0,5 | -0,2 | -0,3 |
| Hs.194654 | BAI1 | 12,7 | 8,5 | 5,8 | 12,1 | 10,4 | 3,7 | 0,2 | 0,1 | -0,2 | -0,2 | -0,3 | -0,1 | -0,7 |
| Hs.514527 | BIRC5 | 700,5 | 597,3 | 598,1 | 532,6 | 610,1 | 509,2 | 0,2 | 0,2 | 0,3 | 0,1 | 0,1 | 0,2 | 0 |
| Hs.74034 | CAV1 | 711,1 | 672 | 667,7 | 713,3 | 4049,2 | 3749,2 | 0,2 | 0 | -2,4 | -2,5 | -0,1 | -0,1 | -2,4 |
| Hs.517356 | COL18A1 | 111,6 | 85,7 | 83,1 | 94,8 | 134,9 | 116 | 0,2 | 0,2 | -0,2 | -0,4 | 0 | -0,1 | -0,6 |
| Hs.40499 | DKK1 | 8340 | 7323,6 | 5600,9 | 6293,2 | 7511,3 | 7648,8 | 0,2 | 0,5 | 0,1 | 0 | 0,2 | -0,2 | -0,4 |
| Hs.586423 | EEF1A1 | 11029,4 | 9798,2 | 9158,5 | 10897,8 | 10380,2 | 11380,8 | 0,2 | 0,2 | 0,2 | -0,2 | -0,2 | -0,3 | -0,2 |
| Hs.644639 | EEF1A1 | 10121,3 | 8704,9 | 8601,4 | 10331,9 | 9248,6 | 11006,7 | 0,2 | 0,2 | 0,1 | -0,2 | -0,3 | -0,3 | -0,1 |
| Hs.586423 | EEF1A1 | 11888,7 | 10484,7 | 10178,2 | 12762,3 | 11087,6 | 12150,2 | 0,2 | 0,2 | 0,1 | -0,2 | -0,3 | -0,3 | -0,2 |
| Hs.488293 | EGFR | 2,6 | 2,7 | 4,2 | 5,2 | 2,2 | 3,1 | 0,2 | -0,1 | 0,1 | -0,4 | -0,4 | 0,1 | -0,1 |
| Hs.488293 | EGFR | 5,6 | 2,7 | 3,2 | 2,5 | 9,4 | 4,6 | 0,2 | 0 | -0,7 | -1 | -0,3 | 0,2 | -0,5 |
| Hs.525600 | HSP90AA1 | 9646,1 | 8562,3 | 8222,4 | 9697,4 | 9100,6 | 9389 | 0,2 | 0,2 | 0,1 | -0,1 | -0,2 | -0,3 | -0,1 |
| Hs.525600 | HSP90AA1 | 10829 | 9825,1 | 9391,1 | 11292 | 10024,8 | 10941,4 | 0,2 | 0,3 | 0,1 | -0,1 | -0,2 | -0,3 | -0,2 |
| Hs.525600 | HSP90AA1 | 11374 | 9739,5 | 9152 | 10855,9 | 10317,8 | 10919,4 | 0,2 | 0,3 | 0,1 | -0,2 | -0,1 | -0,2 | -0,2 |
| Hs.525600 | HSP90AA1 | 11744,3 | 9982,6 | 9397,2 | 11463,3 | 9915,1 | 11344,6 | 0,2 | 0,3 | 0,2 | -0,1 | -0,2 | -0,3 | -0,1 |
| Hs.148741 | IBRDC2 | 3,2 | 3,3 | 1,8 | 1,8 | 2,4 | 3,4 | 0,2 | 0,3 | -0,1 | -0,1 | 0,3 | -0,4 | -0,2 |
| Hs.533782 | KRT8 | 1608,1 | 1499,1 | 1661,4 | 1712,9 | 2085,7 | 2003,1 | 0,2 | -0,1 | -0,3 | -0,5 | -0,3 | -0,1 | -0,3 |
| Hs.132966 | MET | 366,1 | 270 | 250,9 | 179,7 | 373,9 | 382,2 | 0,2 | 0,8 | -0,2 | -0,1 | 0,6 | 0,2 | -1 |
| Hs.372914 | NDRG1 | 745,4 | 693,9 | 771,3 | 788,7 | 2927 | 1872,1 | 0,2 | 0 | -2 | -1,5 | -0,2 | -0,1 | -2 |
| Hs.160953 | P53AIP1 | 22,9 | 23 | 26,7 | 27 | 41,7 | 43,3 | 0,2 | -0,3 | -0,6 | -0,5 | -0,4 | -0,1 | -0,4 |
| Hs.534573 | PML /// LOC161527 /// LOC652671 | 68,3 | 53,9 | 62,8 | 71,9 | 70,8 | 73,9 | 0,2 | -0,1 | -0,2 | -0,6 | -0,3 | 0 | -0,1 |
| Hs.395482 | PTK2 | 78,2 | 72,2 | 70,8 | 72,7 | 110,7 | 109,6 | 0,2 | -0,1 | -0,4 | -0,6 | 0 | 0,2 | -0,2 |
| Hs.523744 | RFWD2 | 374,9 | 311,2 | 352,3 | 323,2 | 272,3 | 269,6 | 0,2 | 0,3 | 0,4 | 0,1 | 0,1 | 0,1 | 0,1 |
| Hs.128856 | SCARA3 | 54,6 | 49,4 | 53,4 | 64,1 | 55,1 | 79,3 | 0,2 | 0 | -0,1 | -0,6 | -0,1 | -0,2 | 0,1 |
| Hs.137569 | TP73L | 4,3 | 7 | 1,3 | 8 | 1,6 | 1 | 0,2 | 2,3 | 2,2 | 1,9 | -0,3 | -2,8 | -0,3 |
| Hs.524368 | VDR | 205,7 | 186,8 | 186,5 | 210,4 | 128,5 | 218,7 | 0,2 | 0,1 | 0,6 | -0,2 | -0,2 | -0,1 | 0,4 |
| Hs.489033 | ABCB1 /// ABCB4 | 1,2 | 2,1 | 5,3 | 19,6 | 37,9 | 19,5 | 0,3 | -2,1 | -3,5 | -3,3 | -3,2 | -1,9 | -3,3 |
| Hs.158932 | APC | 42,8 | 24,6 | 21,1 | 22,9 | 30,7 | 42,9 | 0,3 | 0,3 | 0,1 | -0,2 | 0,4 | 0,3 | -0,6 |
| Hs.631546 | BAX | 50,2 | 35,2 | 56,4 | 75,1 | 43 | 30,8 | 0,3 | -0,1 | 0,5 | 0,4 | -1 | -0,2 | 0,8 |
| Hs.478588 | BCL6 | 42,3 | 34,5 | 35,3 | 48,1 | 8,4 | 28,4 | 0,3 | 0,4 | 2,4 | 0,4 | -0,6 | -0,6 | 1,9 |
| Hs.514527 | BIRC5 | 2020,3 | 1582,1 | 1752,8 | 1527,4 | 1703,3 | 1295,3 | 0,3 | 0,2 | 0,2 | 0,3 | 0,1 | 0,2 | 0 |
| Hs.74034 | CAV1 | 308,5 | 247 | 291,2 | 265,6 | 1679,5 | 1502,8 | 0,3 | 0,2 | -2,3 | -2,6 | 0 | 0 | -2,6 |
| Hs.370771 | CDKN1A | 10,5 | 9 | 4,3 | 12 | 2,2 | 2,2 | 0,3 | 1,5 | 2,8 | 1,7 | 0 | -1,7 | 0 |
| Hs.517356 | COL18A1 | 34,3 | 28,5 | 36,2 | 30,8 | 44,7 | 61,7 | 0,3 | 0,3 | -0,3 | -0,7 | 0 | 0,2 | -0,3 |
|  | EEF1A1 | 11807 | 9019 | 8633,5 | 10682,7 | 9126,3 | 11055,3 | 0,3 | 0,3 | 0,2 | -0,2 | -0,3 | -0,3 | -0,1 |
| Hs.518450 | HD | 24,9 | 19 | 35,9 | 54,9 | 39,8 | 34,8 | 0,3 | 0,1 | -0,2 | -1 | -1,4 | -0,4 | -0,3 |
| Hs.209128 | MAD1L1 | 170,5 | 132,9 | 172,1 | 168 | 112,7 | 117,2 | 0,3 | -0,2 | 0,5 | 0,2 | -0,3 | 0,1 | 0,8 |
| Hs.574741 | NLRC4 | 1,9 | 1,2 | 0,6 | 5,3 | 1,5 | 6,8 | 0,3 | 0,1 | -0,2 | -2,3 | -2,6 | -3,3 | -0,2 |
| Hs.647092 | NOS3 | 144,1 | 94,4 | 146,1 | 138 | 85,7 | 71,3 | 0,3 | -0,3 | 0,5 | 0,6 | -0,3 | 0,2 | 0,7 |
| Hs.349094 | SH2D1A | 2,6 | 2,1 | 3,3 | 1,3 | 3,1 | 6 | 0,3 | 0,1 | -0,2 | -0,3 | -0,1 | -0,1 | -0,3 |
| Hs.192132 | TP73 | 42,5 | 21 | 15,2 | 29,1 | 36,4 | 32,1 | 0,3 | 0,7 | 0,3 | -0,2 | 0 | -0,2 | -0,1 |
| Hs.524368 | VDR | 60,9 | 46,1 | 37,5 | 59,4 | 38,1 | 42,3 | 0,3 | 0,8 | 0,6 | 0,2 | -0,3 | -0,4 | -0,1 |
| Hs.62180 | ANLN | 1689,4 | 1301,5 | 1587,1 | 1345 | 2036,5 | 1142,6 | 0,4 | 0,1 | -0,3 | 0,2 | 0 | 0,3 | -0,3 |
| Hs.62180 | ANLN | 859,3 | 656,4 | 740 | 534,6 | 986,9 | 555,6 | 0,4 | 0,2 | -0,3 | 0,2 | 0,2 | 0,4 | -0,4 |
| Hs.488293 | EGFR | 86,4 | 79,3 | 102 | 59 | 296,1 | 288,6 | 0,4 | 0 | -1,9 | -2,4 | 0,2 | 0,7 | -1,7 |
| Hs.514527 | BIRC5 | 558,7 | 444,1 | 504,1 | 452 | 430,1 | 273,4 | 0,5 | 0,2 | 0,5 | 0,6 | 0 | 0,3 | 0,3 |
| Hs.371147 | THBS2 | 16,7 | 9,1 | 14,2 | 2,3 | 3,4 | 2,2 | 0,5 | 0,3 | 2,3 | 2,1 | 2,2 | 2,7 | 2 |
| Hs.494875 | RGS3 | 153,8 | 77,5 | 119,6 | 102,8 | 112 | 57,2 | 0,6 | 0,3 | 0,2 | 0,6 | -0,1 | 0 | -0,1 |
| Hs.160953 | P53AIP1 | 3,8 | 4 | 5,3 | 5,2 | 10,7 | 3,8 | 0,6 | -0,3 | -1,3 | -0,2 | -0,7 | 0,2 | -1,3 |
| Hs.526464 | PML | 59,6 | 47,1 | 41,3 | 34,1 | 33,9 | 28,6 | 0,6 | 0,9 | 0,9 | 0,1 | -0,2 | -0,6 | 0 |
| Hs.148741 | IBRDC2 | 2,2 | 2,7 | 7,5 | 5,4 | 9,9 | 2 | 0,7 | -1,7 | -3,1 | 0,3 | -0,6 | 0,8 | -0,8 |
| Hs.2490 | CASP1 | 2,8 | 1,9 | 1,4 | 0,7 | 4,4 | 0,8 | 0,8 | 1,1 | -0,1 | 1,3 | 0,8 | 0,2 | -2,7 |
| Hs.656 | CDC25C | 270,2 | 161,4 | 248,7 | 211,6 | 314 | 160,4 | 0,8 | 0,3 | -0,1 | 0,1 | 0,1 | 0,5 | -0,4 |
| Hs.656 | CDC25C | 93,3 | 48,6 | 58,3 | 51,3 | 99,8 | 61,6 | 0,8 | 0,2 | -0,4 | -0,3 | -0,1 | 0,6 | -0,7 |
| Hs.171695 | DUSP1 | 43,9 | 17 | 22,9 | 25,1 | 6,3 | 14,3 | 0,8 | 0,8 | 3,1 | 0,3 | -0,1 | -0,1 | 1,7 |
| Hs.145269 | TNFRSF10C | 27,8 | 14,9 | 19 | 20,9 | 4 | 3,2 | 1,1 | 0,6 | 2,3 | 1,7 | -1 | -0,1 | 2,1 |
| Hs.160953 | P53AIP1 | 6 | 2,5 | 2,2 | 2,4 | 11,9 | 4,3 | 1,2 | 1,3 | -0,6 | -0,1 | 0,1 | 0,1 | -2,1 |
| Hs.510409 | CCNK | 12,6 | 7,6 | 8,8 | 14,3 | 5,3 | 5,9 | 1,3 | 0,9 | 1,2 | -0,5 | -1,5 | -1,2 | 0 |
| Hs.145269 | TNFRSF10C | 15,1 | 5 | 2,8 | 1,5 | 1,8 | 1,2 | 1,3 | 1,9 | 2,3 | 1,9 | 1,5 | 0 | 0,5 |
| Hs.160953 | P53AIP1 | 10,5 | 1,2 | 3,5 | 2,3 | 24,4 | 4,3 | 1,5 | 1 | -1,2 | -0,3 | -0,5 | -0,1 | -2,4 |
| Hs.137569 | TP73L | 25,3 | 7,9 | 10,4 | 6,7 | 4,2 | 22 | 1,5 | 1,1 | 2,7 | -0,8 | 0,6 | 0 | 1,8 |
| Hs.489033 | ABCB1 | 9,3 | 2,3 | 8,8 | 18,3 | 27,9 | 31,5 | 1,6 | -0,2 | -1,6 | -3,2 | -3 | -0,6 | -1,8 |
| Hs.488293 | EGFR | 21 | 7,7 | 18,6 | 21,5 | 26,5 | 23,6 | 1,6 | 0,4 | -0,5 | -1,9 | -1,3 | -0,1 | -0,5 |
| Hs.2490 | CASP1 | 7,2 | 1,6 | 0,7 | 2,9 | 0,9 | 1,7 | 1,8 | 2,4 | 2,9 | -0,3 | -1,3 | -1,7 | 0,3 |
| Hs.513617 | MMP2 | 40,2 | 10,8 | 21,5 | 26,7 | 31,7 | 20,9 | 1,9 | 0,6 | 0,6 | -0,1 | -1,4 | -0,2 | -0,5 |
| Hs.499094 | PYCARD | 13,7 | 3,8 | 1,8 | 21,9 | 1,4 | 3,3 | 2,3 | 2,4 | 2,2 | -0,2 | -3,1 | -3,6 | -0,3 |
| Hs.396530 | HGF | 5,6 | 0,2 | 0,9 | 0,6 | 0,6 | 0,8 | 2,4 | 3,1 | 3,2 | -0,2 | 0,3 | 0,2 | -0,5 |
| Hs.521181 | IRF5 | 32,9 | 10,9 | 4,3 | 30,9 | 29,1 | 7 | 2,5 | 3,3 | 0,4 | 0,3 | -1,7 | -2,7 | -2,6 |
| Hs.489033 | ABCB1 | 12,4 | 1,7 | 12,7 | 6,3 | 12,6 | 12,1 | 2,6 | -0,1 | -0,1 | -2,7 | -2,4 | 0,9 | -0,3 |
| Hs.348365 | CASP1 /// COP1 | 7,2 | 0,6 | 4,3 | 5,1 | 8,7 | 1,8 | 2,7 | 0,4 | -0,3 | 0,2 | -2,4 | 0 | -0,7 |
| Hs.526464 | PML | 43,6 | 4,2 | 40,3 | 5,6 | 4,9 | 4,6 | 2,8 | 0 | 2,7 | -0,4 | -0,3 | 1,9 | 2,2 |
| Hs.349094 | SH2D1A | 8,9 | 1,7 | 2,6 | 4,2 | 1,3 | 1,2 | 2,8 | 1,8 | 2,5 | 0 | -2,1 | -1,9 | -0,2 |
| Hs.137569 | TP73L | 13,9 | 0,6 | 15,6 | 19,3 | 3,3 | 4,7 | 2,8 | -0,4 | 1,4 | -2,1 | -3,4 | -0,3 | 2,3 |
| Hs.520421 | PERP | 12,2 | 1,3 | 0,7 | 0,9 | 1,6 | 0,9 | 3,6 | 4,1 | 3,2 | 0,1 | -0,2 | 0,6 | -0,6 |
